# Gut microbiome-derived glycine lipids are diet-dependent modulators of hepatic injury and atherosclerosis

**DOI:** 10.1016/j.jlr.2022.100192

**Published:** 2022-03-10

**Authors:** Courtney L. Millar, Liya Anto, Chelsea Garcia, Mi-Bo Kim, Anisha Jain, Anthony A. Provatas, Robert B. Clark, Ji-Young Lee, Frank C. Nichols, Christopher N. Blesso

**Affiliations:** 1 Department of Nutritional Sciences, University of Connecticut, Storrs, CT, USA; 2 The Marcus Institute for Aging Research, Harvard Medical School, Boston, MA, USA; 3 Center for Environmental Sciences and Engineering, University of Connecticut, Storrs, CT, USA; 4 Department of Immunology, UConn Health, Farmington, CT, USA; 5 Department of Medicine, UConn Health, Farmington, CT, USA; 6 Department of Periodontology, UConn Health, Farmington, CT, USA

**Keywords:** liver, dietary fat, cholesterol, heart, inflammation, high-fat diet, L654, L567, alanine aminotransferase, mouse models, Bacteroidetes, Bacteroidota, ALT, alanine aminotransferase, CCL2, C-C motif chemokine ligand 2, cDNA, complementary DNA, ESI, electrospray ionization, HFD, high-fat diet, IL-1β, interleukin 1-beta, L342, lipid 342, L430, lipid 430, L567, lipid 567, L654, lipid 654, L1256, lipid 1256, LPS, lipopolysaccharide, MHC, major histocompatibility complex, MRM, multiple reaction monitoring, NEFAs, non-esterified fatty acids, PGE_2_, prostaglandin E_2_, qT–PCR, quantitative RT-PCR, RNA-Seq, RNA sequencing, SAA, serum amyloid A, TG, triglyceride, TLR, Toll-like receptor, UPLC, ultraperformance liquid chromatography

## Abstract

Oral and gut Bacteroidetes produce unique classes of serine-glycine lipodipeptides and glycine aminolipids that signal through host Toll-like receptor 2. These glycine lipids have also been detected in human arteries, but their effects on atherosclerosis are unknown. Here, we sought to investigate the bioactivity of bacterial glycine lipids in mouse models of atherosclerosis. Lipid 654 (L654), a serine-glycine lipodipeptide species, was first tested in a high-fat diet (HFD)-fed *Ldlr*^−/−^ model of atherosclerosis. Intraperitoneal administration of L654 over 7 weeks to HFD-fed *Ldlr*^−/−^ mice resulted in hypocholesterolemic effects and significantly attenuated the progression of atherosclerosis. We found that L654 also reduced liver inflammatory and extracellular matrix gene expression, which may be related to inhibition of macrophage activation as demonstrated in vivo by lower major histocompatibility complex class II gene expression and confirmed in cell experiments. In addition, L654 and other bacterial glycine lipids in feces, liver, and serum were markedly reduced alongside changes in Bacteroidetes relative abundance in HFD-fed mice. Finally, we tested the bioactivities of L654 and related lipid 567 in chow-fed *Apoe*^−/−^ mice, which displayed much higher fecal glycine lipids relative to HFD-fed *Ldlr*^−/−^ mice. Administration of L654 or lipid 567 for 7 weeks to these mice reduced the liver injury marker alanine aminotransferase, but other effects seen in *Ldlr*^−/−^ were not observed. Therefore, we conclude that conditions in which gut microbiome-derived glycine lipids are lost, such as HFD, may exacerbate the development of atherosclerosis and liver injury, whereas correction of such depletion may protect from these disorders.

Driven by recent advancements in high-throughput sequencing technology, there has been a dramatic increase in research supporting associations between the gut microbiome, diet, and metabolic diseases. The Bacteroidetes and Firmicutes phyla typically comprise more than 90% of the bacteria found in the human gut microbiome (1). Some studies report obese mice, and humans have an increased relative abundance of fecal Firmicutes and a lower abundance of Bacteroidetes compared with lean controls (2, 3). Lower microbial diversity and a higher Firmicutes/Bacteroidetes ratio often characterize the gut dysbiosis seen in high-fat diet (HFD)-induced obesity and metabolic diseases (4, 5, 6, 7, 8). Even though a higher prevalence of Bacteroidetes appears to be associated with beneficial health outcomes, some species of the phylum, such as *Porphyromonous gingivalis*, can invade the endothelial layer of arteries and translocate to the circulatory system, allowing for potential inflammatory signaling (9). Alongside changes in gut microbiota composition and translocation of whole microbes, exposure to microbiota-produced metabolites and bioactive compounds may also drive disease development. Thus, characterizing the host response to metabolic products of Bacteroidetes is considered critical for the understanding of gut microbiome-host interactions in human metabolic diseases (10).

Besides the well-known role of lipopolysaccharide (LPS) in Toll-like receptor (TLR) activation, emerging classes of gut microbiota-derived lipids relevant to inflammation-related disease are the glycine lipids first identified in *P. gingivalis* and found to be broadly expressed in the Bacteroidetes phylum (11, 12, 13). These unique classes of bacterial lipids signal through TLR2 and include the glycine amino lipid species, lipid 342 (L342) and lipid 567 (L567) (12), named for their negative ion mass, as well as the serine-glycine lipodipeptides, lipid 430 (L430), lipid 654 (L654), and lipid 1256 (L1256) (Fig. 1A) (11, 13). These glycine lipids are related to each other through the constituent core L342 structure consisting of a 3-OH *iso* C_17:0_ fatty acid, which is amide linked to a glycine (12). The addition of an ester-linked fatty acid (*iso* or *anteiso* C_15:0_) to L342 yields the other glycine amino lipid species, L567 (12). L654 consists of a terminal serine that is amide linked to the glycine of L567, thus making it a serine-glycine lipodipeptide (12). Removal of the ester-linked fatty acid from L654 produces L430, which could occur via phospholipase A2-mediated hydrolysis in humans (14). L654 can be modified further through the attachment of a diacylated phosphoglycerol to its serine moiety to form L1256 (13). The synthesis and bioactivities of these related glycine lipids are still being resolved, although they are likely shed from bacteria through outer membrane vesicles or cell turnover, and have been shown to activate TLR2 in vitro (11, 12, 13, 15), and induce acute inflammation in mice via TLR2 (11). Notably, it was recently reported that some of these glycine lipids are found in human serum, brain, and carotid artery atheroma (14). Because of the localization of these lipids in atherosclerotic lesions and signaling through inflammatory TLRs, it is thought that these bacterial-derived lipids may contribute to the pathogenesis of atherosclerosis (9, 14). Furthermore, it seems plausible that dietary factors known to influence the relative abundance of Bacteroidetes (e.g., dietary fat) may influence host exposure to these bacterial glycine lipids. Thus, we sought to investigate the bioactivities of bacterial glycine lipids in mouse models of atherosclerosis and characterize the glycine lipid content in the feces of mice fed different diets. We hypothesized that chronic exposure to glycine lipids would exacerbate atherosclerosis progression through increasing inflammation.

**Fig. 1:**
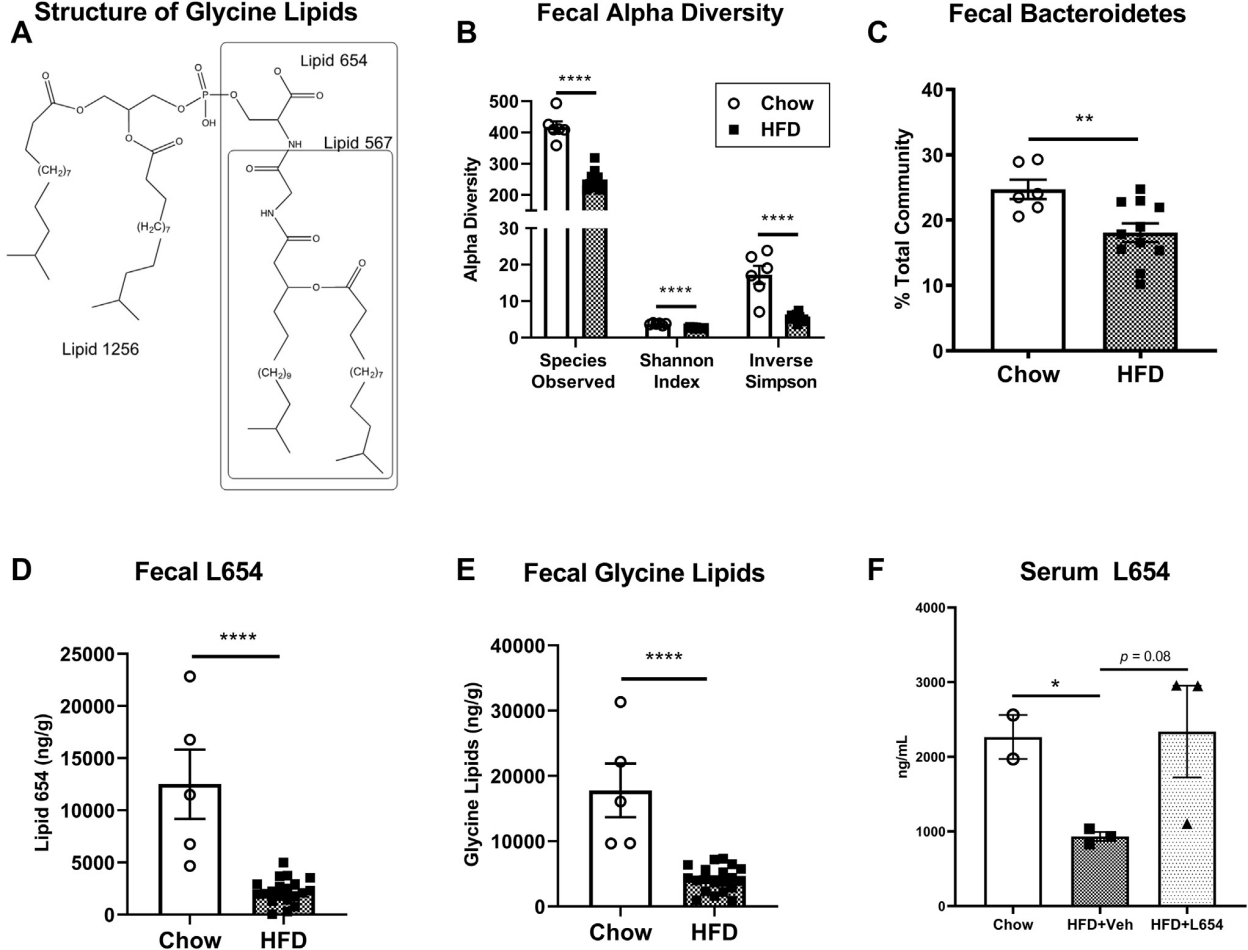
Western-type HFD markedly decreases fecal bacterial glycine lipids and fecal microbiota diversity. Structures of bacterial glycine lipids used for experiments (L567, L654, and L1256) depicting the dominant lipid species within each lipid class (A). Cecal feces were aseptically collected for characterization of 16S V4 region (B, C). Measures of fecal microbiota alpha diversity (B) and fecal Bacteroidetes (C) of *Ldlr*^−/−^ mice housed in different cages and fed either a standard low-fat chow diet or Western-type HFD for 14 weeks (*n* = 6–11, mean ± SEM). Lipids were extracted from feces and L654 (D), and total bacterial glycine lipids (E) were quantified by UPLC-MS/MS (C, D) (*n* = 5–20, mean ± SEM). Serum L654 was quantified by UPLC-MS/MS after pooling three individual animals per sample (*n* = 2–3 pooled, mean ± SEM). Statistical significance determined by two-tailed Student’s *t-*test (∗*P* < 0.05, ∗∗*P* < 0.01, and ∗∗∗∗*P* < 0.0001).

## Materials and methods

### Bacterial glycine lipid extraction and HPLC fractionation

Bacterial glycine lipids (L1256, L654, and L567) were extracted from *P. gingivalis* (ATCC 33277, type strain), fractionated using normal-phase semipreparative HPLC, and fractions dried under nitrogen as previously described (12, 13). Lipid fractions containing L1256, L654, or L567 were pooled by lipid class, and pooled fractions were individually refractionated using the same normal-phase HPLC with solvent containing 0.1% acetic acid. Fractions were evaluated by LC-MS (see later), and fractions containing either L1256, L654, or L567 were pooled by lipid class, and sample purity was evaluated using LC-MS. L1256, L654, and L567 were evaluated using ultraperformance liquid chromatography (UPLC)-QTOF LC-MS/MS (Sciex500). L567 and L654 were shown to contain l-serine (11) and (*R*)-3-OH isobranched C_17:0_ (14). L567 and L654 structures were previously verified by total synthesis by Dietz *et al.* (16) and Nichols *et al.* (12), respectively. NMR verification of L654 and L1256 structures was reported previously by Clark *et al.* (11) and Nichols *et al.* (13), respectively. High-resolution mass spectra revealed negative ion masses of *m/z* 1255.9337 for L1256, *m/z* 653.5060 for L654, and *m/z* 566.4748 for L567. Sonicated lipid preparations were used in cell experiments and animal studies, with vehicle control solutions undergoing the same sonication procedures as bacterial lipid solutions. The structures of glycine lipids used for experiments are shown in Fig. 1A.

### Animals and diets

Male *Ldlr*^−/−^ mice and *Apoe*^−/−^ mice were obtained from Jackson Laboratory (Bar Harbor, ME) and allowed to acclimate for 2 weeks prior to the start of experiments. We chose to use male mice for these initial studies as they are more responsive to HFD-induced metabolic dysfunction than female mice. In a first study, we investigated the effects of L654 on disease progression in *Ldlr*^−/−^ mice. Male *Ldlr*^−/−^ mice (6 weeks of age) were randomized into three experimental groups: a chow diet-fed reference group, a bacterial L654-injected HFD-fed group (HFD + L654), or a vehicle-injected HFD-fed control group (HFD + Veh). The chow group was maintained on a standard low-fat chow diet for 14 weeks to serve as a reference group for disease progression and fecal analyses. To induce hypercholesterolemia, the HFD groups were fed ad libitum a base HFD, which consisted of 45% kcal from fat, 34% sucrose, and 0.15% added cholesterol (0.2% cholesterol by weight in total), for a total of 14 weeks (supplemental Table S1). Body weight and food intake were assessed weekly. Mice were provided with fresh food weekly. Following 7 weeks on HFD, both groups received intraperitoneal injections of their respective sonicated solutions (vehicle or L654) every 48 h for the remaining 7 weeks. The vehicle consisted of sterile saline with 0.03% v/v ethanol. L654 was administered at 1 μg dose with each injection, a dose that was shown to acutely increase serum C-C motif chemokine ligand 2 (CCL2) in a TLR2-dependent manner with a single intraperitoneal injection in mice (11).

In a second study, we examined effects of short-term HFD feeding on fecal glycine lipid content of *Apoe*^−/−^ mice. Male *Apoe*^−/−^ mice (8 weeks of age) were allowed to acclimate for 2 weeks on low-fat chow diets prior to the feeding of HFD for an additional 2 weeks. Fecal samples were collected at the end of consuming chow and HFD for 2 weeks.

In a third study, we investigated the effects of L654 and L567 on atherosclerosis in chow-fed *Apoe*^−/−^ mice. About 13-week-old male *Apoe*^−/−^ mice were maintained on a low-fat chow diet while being intraperitoneally injected every 48 h with either L654 (1 μg), L567 (1 μg), or vehicle control for 7 weeks. After their respective diets and/or treatments, the mice were fasted for 6–8 h prior to being anesthetized with ketamine/xylazine (100 mg/kg ketamine and 10 mg/kg xylazine) followed by blood collection by cardiac puncture following euthanasia. Blood samples were allowed to clot at room temperature for 30 min before serum was isolated by centrifugation (10,000 *g* for 10 min at 4°C) and then stored at −80°C. Animals were perfused with sterile saline to clear any residual blood from tissues, which may interfere with analyses. Fecal samples were collected and snap-frozen before storage at −20°C. Tissues were harvested, weighed, snap-frozen in liquid nitrogen, and then stored at −80°C. Hearts were processed for aortic sectioning and subsequently stored at −80°C prior to further analysis as previously described (17). Saline-perfused liver sections (left lateral lobe) were fixed in 10% neutral-buffered formalin for 48 h. All mice were housed in a temperature-controlled room and maintained in a 14 h light/10 h dark cycle at the University of Connecticut-Storrs vivarium. All procedures proposed in this study have been approved by the Animal Care and Use Committee of the University of Connecticut-Storrs. All animal experiments were in accordance with the Guide for the Care and Use of Laboratory Animals published by the National Institutes of Health (18).

### Serum biochemical analysis

Total serum cholesterol, HDL-C, triglycerides (TGs), and non-esterified fatty acids (NEFAs) were measured using kits purchased from Wako Diagnostics (Richmond, VA). In addition, glucose, alanine aminotransferase (ALT), and aspartate aminotransferase were measured by enzymatic methods following kit instructions (Pointe Scientific, Inc, Canton, MI). Inflammatory markers serum amyloid A (SAA), CCL2, and interleukin 1-beta (IL-1β) were measured in serum by indirect sandwich ELISAs using recommended dilutions (R&D Systems, Minneapolis, MN).

### Atherosclerosis analysis

Mouse hearts were prepared and sectioned to isolate the aortic root as previously described (17). In brief, hearts were sectioned, and slides were stained with Oil Red O. Each section was measured for total lesion area by an observer blinded to group assignment.

### Liver RNA isolation, complementary DNA synthesis, and real-time quantitative RT-PCR

Total RNA was isolated from the liver using TRIzol (Life Technologies, Carlsbad, CA, USA), treated with DNAse I, and reverse transcribed to complementary DNA (cDNA). Total RNA was treated with DNase I and reverse transcribed using iScript cDNA synthesis kit (Bio-Rad, Hercules, CA). Real-time quantitative RT-PCR (qRT-PCR) was performed using iTaq Universal SYBR Green Supermix (Bio-Rad) on a CFX96 real-time-PCR detection system (Bio-Rad). Gene expression was normalized to the geometric mean of the reference genes, glyceraldehyde 3-phosphate dehydrogenase (*Gapdh*), β-actin (*Actb*), and ribosomal protein, large, P0 (*Rplp0*) for liver using the 2^−ΔΔCt^ method. See supplemental Table S2 for primer sequences used.

### Transcriptomics analysis of mouse liver tissue

RNA was isolated from snap-frozen liver tissues from Western-type diet-fed *Ldlr*^−/−^ mice injected with vehicle or L654 (1 μg every 48 h) for 7 weeks (*n* = 8 per group). For transcriptome analysis, sample preparation and library constructions were performed at the UConn Center for Genome Innovation and then sent to Psomagen, Inc (Rockville, MD) for next-generation RNA sequencing (RNA-Seq). In brief, after performing quality control, qualified samples then proceeded to library construction. The sequencing library was prepared by random fragmentation, followed by 5′ and 3′ adapter ligation. Adapter-ligated fragments were then PCR amplified and gel purified. For cluster generation, the library was loaded into a flow cell where fragments were captured on a lawn of surface-bound oligos complementary to the library adapters. Each fragment was then amplified into distinct and clonal clusters through bridge amplification. When cluster generation was complete, the templates were ready for sequencing. Twenty million reads were then obtained using a paired-end 150 bp module on Illumina platform. Sequencing data were then converted into raw data for the analysis. The Illumina sequencer raw images were converted into FASTQ utilizing Illumina package bcl2fastq. Next, bioinformatics analysis on RNA-Seq data was conducted at the UConn Computational Biology Core. Adapter sequences were trimmed from reads with Trimmomatic. Read sequences were then aligned to the mouse reference genome. The number of fragments mapping to each annotated gene were counted using HTSeq. Differential expression analysis of count data with DESeq2 was then conducted. For each gene, *P* values were calculated by asking if the log2 fold change for treatment/control was significantly different from zero, and adjusted *P* values were calculated to account for multiple testing by Benjamini-Hochberg method. Excluded from the analysis were genes with less than 20 fragments mapping across all samples because there is little power to assess expression differences. Genes with extreme outliers, identified by Cook’s distance, were not given *P* values. Gene set enrichment analysis was then conducted using gProfiler (https://biit.cs.ut.ee/gprofiler/gost ) to examine gene ontology.

### Liver lipid extraction and tissue histology

Liver lipids were extracted using methods previously described (19) with a modified Folch method. Following extraction and solubilization in 1% Triton X-100, total cholesterol, free cholesterol, and TGs were measured using enzymatic kits (Wako Diagnostics, Richmond, VA). Cholesteryl esters were also calculated as (total cholesterol − free cholesterol) × 1.67.

For liver histology, fresh tissue sections were placed in cassettes and fixed in 10% neutral-buffered formalin. Samples were processed and stained with hematoxylin and eosin at the Connecticut Veterinary Medical Diagnostic Laboratory in Storrs, CT, as previously reported (19). Slides were imaged and used to present representative images of the tissue histology in each group.

### Gut microbiota analysis

Cecal feces samples were aseptically harvested from separately housed mice (*n* = 6–11/group) and submitted to the University of Connecticut-Storrs Microbial Analysis, Resources and Services facility for microbiota characterization using 16S V4 analysis as previously described (20). Following DNA extraction and amplification, PCR products were pooled for quantification, normalized, and cleaned prior to clustering into operational taxonomic units (20).

### Bacterial glycine lipid treatment of RAW264.7 macrophages

Murine RAW264.7 macrophages were obtained from ATCC (Manassas, VA) and cultured in a humidified incubator at 37°C and 5% CO_2_. Cells were maintained in DMEM (4 g/l glucose) containing sodium pyruvate, 10% fetal bovine serum (Hyclone, Logan, UT), 2 mM l-glutamine, 100 U/ml penicillin, 100 μg/ml streptomycin antibiotic (Thermo Fisher Scientific, Waltham, MA), and 100 μg/ml normocin (Invitrogen, Carlsbad, CA). Cell counts and viability were routinely measured using Trypan blue and TC-20 automated cell counter (Bio-Rad).

In the first set of cell experiments, RAW264.7 macrophages were incubated with L654 (0.5, 1, and 2 μg/ml), 100 ng/ml LPS (*Escherichia coli* 0111:B4) (Sigma-Aldrich, St. Louis, MO), or vehicle control for 24 h and then stimulated with LPS (100 ng/ml) for another 8 h. Conditioned media and RNA were collected for prostaglandin E_2_ (PGE_2_) ELISA (Arbor Assays, Ann Arbor, MI) and qRT-PCR, respectively. In the second set of cell experiments, RAW264.7 macrophages were treated with L654 (0.5, 1, and 2 μg/ml), L1256 (0.5, 1, and 2 μg/ml), or vehicle control for 24 h and then stimulated with recombinant murine IFNγ (Peprotech, East Windsor, NJ) for another 24 h in the presence or the absence of the bacterial lipids. Sonicated bacterial lipid preparations were added to culture dishes to achieve the desired concentrations. RNA was isolated, and cDNA was synthesized and used for qRT-PCR to measure gene expression. Macrophage mRNA was standardized to Gapdh expression using the 2^−ΔΔCt^ method.

### Quantification of bacterial glycine lipids and neutral sterols

For fecal analysis, 100 mg of the samples were weighed and then homogenized with 4 ml of a 1.33:2.67:1 chloroform:methanol:water solution. Following vigorous vortexing, 1.5 ml of chloroform and 1.5 ml of solution that consisted of 2 M potassium chloride and 0.5 M dipotassium phosphate. Samples were then vortexed and centrifuged at 2,000 rpm for 10 min at 20°C. The lower organic phase was removed and dried under nitrogen gas. A silica solid-phase extraction cartridge (Biotage Isolute, Uppsala, Sweden) was conditioned with 4 ml of chloroform. Next, the total dried lipid was dissolved in 0.5 ml of chloroform and spiked with 10 μl of L654 (from a 20 μg/ml stock). Lipid samples were loaded onto the cartridge, and the drained liquid was collected. Four milliliters of chloroform was added to the cartridge to elute the neutral lipid fraction. This was followed with an addition of 5 ml of 9:1 acetone:methanol to elute the glycolipid fraction. Finally, 5 ml of methanol was added to the cartridge to elute the phospholipid fraction. All solvents were evaporated off under nitrogen gas and reconstituted with 200 μl of methanol. Samples were transferred and filtered with a syringe into a 300 μl liquid chromatography vial. Then samples were injected and analyzed using an UPLC coupled with a tandem quadrupole mass spectrometer (MS/MS). Quality control and actual samples were analyzed using a Waters Acquity UPLC coupled with an Acquity TQD tandem mass spectrometer (Waters Co, Milford, MA) at the UConn Center for Environmental Sciences and Engineering Analytical Facility. An Acquity UPLC CSH C18 (1.7 μm, 2.1 × 100 mm) column, heated to 50°C and with a sample injection volume of 20 μl on a 20 μl loop, was utilized for analyte separation. The mobile phase consisted of 10 mM ammonium formate in 40% water/60% acetonitrile (solvent A), and 10 mM ammonium formate in 90% isopropyl alcohol/10% acetonitrile (solvent B) was employed for gradient elution. Initial flow of 75% solvent A was held for 3.0 min before increasing linearly to 100% solvent B until 8.0 min, after which the column was reconditioned to initial state for another 1.0 min. Total run time was 9.0 min with a constant flow rate of 0.4 ml/min. The detection and quantification of analytes and surrogate compound were performed in negative electrospray ionization (ESI)-MS/MS mode (multiple reaction monitoring [MRM]) using the Waters, Inc IntelliStart software for analyte signal optimization. Statistical analysis for obtaining calibration and quantification results for all compounds was performed using Waters QuanLynx, which is included in the MassLynx software version 4.2 (Waters Co). Parameters for the mass spectrometer were set as follows: capillary voltage, 2.0 kV; cone voltage, 30V; desolvation temperature, 400°C; source temperature, 120°C; desolvation gas flow, 750 l/h; and collision gas flow, 0.2 ml/min.

Serum, tissue, and additional fecal samples were extracted and analyzed as follows. Aorta samples were minced with a scalpel and transferred to glass tubes for lipid extraction. Fecal, serum, and tissue samples were first extracted using the method on Bligh and Dyer (shown previously) followed by acidification with acetic acid and re-extraction of the samples with chloroform (2 ml). The neutral and acidic extracts were combined for each sample and dried. Each lipid sample was dissolved in chloroform, and an aliquot was dried and weighed using a Cahn Electrobalance. The lipids were then supplemented with internal standard, and serine/glycine lipids were evaluated using UPLC-QTOF LC-MS (Sciex500) and MRM. Lipids were quantified using a Sciex500 LC-MS equipped with the same column and using the same UPLC solvent gradient as used for the Waters Acquity UPLC/Acquity TQD tandem mass spectrometer. All lipids were detected using the characteristic negative ion transitions determined in MS/MS mode. Quantification of lipid analytes was performed in negative ESI-MS/MS mode (MRM).The following were the parameters for the Sciex500 QTOF TurbolonSpray: curtain gas, 30; ion source gas 1 at 55 and ion source II at 60; and temperature of 500^°^C. The declustering potential was −80 V, and the collision energy ranged from −8 to −60 V. Statistical analysis for quantification of results for all compounds was performed using the Sciex500 software.

The neutral sterols cholesterol, coprostanol, and coprostanone were also measured in feces of mice, isolated in the neutral lipid fraction derived as described previously. Quality control and actual samples were injected and analyzed using the Acquity UPLC-MS/MS system described previously. An Acquity UPLC BEH C18 (1.7 μm, 2.1 × 50 mm) column, maintained at 25°C and with a sample injection volume of 5 μl on a 20 μl loop, was utilized for analyte separation. The mobile phase consisted of 0.1% formic acid in 100% water (solvent A), and 0.1% formic acid in 100% acetonitrile (solvent B) was employed for isocratic elution. Initial flow of 5% solvent A was held for a total run time of 5 min with a constant flow rate of 0.7 ml/min. The detection and quantification of analytes were performed in positive ESI-MS/MS mode (MRM) using the Waters, Inc IntelliStart software for analyte signal optimization. Statistical analysis for obtaining calibration and quantification results for all compounds was performed using Waters QuanLynx, which is included in the MassLynx software, version 4.2. Parameters for the mass spectrometer were set as follows: capillary voltage, 3.2 kV; cone voltage, 40 V; desolvation temperature, 350°C; source temperature, 125°C; desolvation gas flow, 600 l/h; and collision gas flow, 0.2 ml/min.

### Statistical analysis

For comparison of two groups, significance was determined using a two-tailed Student’s *t* test. For comparison of more than two groups, a one-way ANOVA was performed as appropriate with multiple comparisons using Fisher’s least squares difference test. A *P* value <0.05 was considered statistically significant. Bivariate Spearman correlations were used to assess relationships between variables. All statistical analyses were conducted using GraphPad Prism, version 8 software. Data are reported as mean ± SEM.

## Results

### Bacterial glycine lipids are reduced in feces and serum by Western-type HFD in Ldlr^−/−^ mice and are associated with lower fecal Bacteroidetes and higher body weight

To study the effects of diet on bacterial glycine lipid exposure and the effect of L654 on atherosclerosis, we first fed male *Ldlr*^−/−^ mice (aged 6 weeks) a Western-type HFD or low-fat chow diet for 14 weeks. During the last 7 weeks of the HFD, mice were intraperitoneally injected with L654 (1 μg every 48 h) (HFD + L654) or vehicle control (HFD + Veh). Following 14 weeks of Western-type HFD, both HFD groups weighed significantly more than the chow-fed mice as expected (Table 1). There were no differences in body weight or food intake between the two groups of mice fed HFD. However, epididymal fat mass and percent of epididymal fat relative to body weight were significantly lower (−27% and −23%, respectively) in the L654-treated mice when compared with HFD + Veh (Table 1).

**Table 1:** Food intake and body and tissue weights of *Ldlr*^−/−^ mice after 14 weeks of diets

Variable	Chow	HFD + vehicle	HFD + L654
Body weight (g)	31.7 ± 1.34	39.8 ± 1.00^a^	37.4 ± 1.33^a^
Food intake (g/day)	—	3.50 ± 0.074	3.67 ± 0.072
Liver weight (g)	1.35 ± 0.04	2.18 ± 0.16^a^	1.86 ± 0.12^a^
% Liver	4.28 ± 0.12	5.40 ± 0.27^a^	4.92 ± 0.19^a^
Epididymal adipose (g)	0.87 ± 0.25	2.32 ± 0.15^a^	1.69 ± 0.20^a^^,^^b^
% Epididymal adipose	2.54 ± 0.65	5.77 ± 0.27^a^	4.34 ± 0.43^a^^,^^b^

Values are mean ± SEM for all groups (Chow, *n* = 9/group; HFD + vehicle and L654, *n* = 15/group).

a Indicates *P* value < 0.05 versus chow reference group.

b Indicates *P* value < 0.05 versus HFD + vehicle control.

As expected, HFD feeding significantly modulated gut microbiota diversity measures and taxa relative abundances compared with chow-fed mice. Mice fed the HFD (i.e., both HFD + Veh and HFD + L654) had significantly reduced measures of alpha diversity compared with chow-fed mice (Fig. 1B). HFD resulted in a significantly reduced relative abundance of the Bacteroidetes phylum (−27%; Fig. 1C). Compared with the chow group, mice fed HFD also had increased relative abundance of fecal Proteobacteria (+100%) and an increased Firmicutes/Bacteroidetes ratio (+52%) (supplemental Fig. S1). The observed reductions in bacterial diversity, as well as increases in both Proteobacteria and the Firmicutes/Bacteroidetes ratio, were consistent with the induction of obesity-related gut dysbiosis in these animals (21). Notably, there were also significant reductions in fecal L654 (−82%) and total fecal glycine lipids (−76%) in HFD-fed mice compared with chow-fed animals (Fig. 1D,E). Fecal L654 (*r* = −0.68, *P* = 0.009) and total fecal glycine lipids (*r* = −0.53, *P* = 0.044) were inversely associated with end point body weights of animals (supplemental Fig. S2A,B). In contrast, fecal L654 (*r* = 0.74, *P* = 0.045) and total fecal glycine lipids (*r* = 0.80, *P* = 0.014) were positively associated with fecal Bacteroidetes (supplemental Fig. S2C,D). There were no differences between the HFD + L654 and HFD + Veh groups in fecal bacterial diversity (supplemental Fig. S3), phyla composition (supplemental Fig. S4A), Firmicutes/Bacteroidetes ratio (supplemental Fig. S4B), or fecal glycine lipids (supplemental Fig. S4C). In line with fecal changes, both L654 (−59%) (Fig. 1F) and total glycine lipids (−76%) (supplemental Fig. S5A) were reduced in pooled serum from HFD + Veh compared with low-fat chow diet. The intraperitoneal injection of L654 partially corrected for the depletion of circulating L654 seen with HFD, as L654 tended to be higher in pooled serum from HFD + L654 compared with HFD + Veh (*P* = 0.085).

### Chronic intraperitoneal administration of L654 has hypolipidemic effects in Ldlr^−/−^ mice

As anticipated, the mice fed HFD (both HFD + Veh and HFD + L654) for 14 weeks were hyperlipidemic compared with the chow-fed mice, increasing serum total cholesterol up to 6-fold (Table 2). However, the intraperitoneal injection of L654 for the final 7 weeks of HFD resulted in significantly lower serum total cholesterol (−24%) and non-HDL-C (−25%) in HFD + L654 mice compared with vehicle injection (HFD + Veh) (Table 2). In addition, L654-injected mice experienced a significant reduction in serum NEFAs (−14%) compared with the vehicle-injected animals (Table 2). However, there were no significant effects of L654 injections on serum HDL-C, TGs, glucose, homeostasis model assessment of insulin resistance, or a selection of inflammatory markers (SAA, CCL2, and IL-1β) (Table 2).

**Table 2:** Serum biochemical analysis of *Ldlr*^−/−^ mice

Variable	Chow	HFD + vehicle	HFD + L654
Total cholesterol (mg/dl)	289.5 ± 21.2	1,701 ± 164^a^	1,298 ± 109.8^a^^,^^b^
HDL-C (mg/dl)	—	55.8 ± 3.4	57.7 ± 2.0
Non-HDL-C (mg/dl)	—	1,645 ± 162.5	1,240 ± 109.7^b^
TGs (mg/dl)	328.4 ± 34.8	658.2 ± 117.8^a^	449.0 ± 61.2
NEFAs (mmol/l)	0.49 ± 0.03	0.42 ± 0.03	0.36 ± 0.01^a^^,^^b^
Glucose (mg/dl)	144.2 ± 13.9	174.0 ± 7.2^a^	168.9 ± 6.2
Insulin (ng/ml)	—	0.32 ± 0.03	0.28 ± 0.04
HOMA-IR	—	3.09 ± 0.38	2.57 ± 0.34
SAA (ng/ml)	—	8,166 ± 936	6,273 ± 1,270
CCL2 (pg/ml)	—	126.3 ± 13.8	125.3 ± 18.2
IL-1β (pg/ml)	—	115.4 ± 15.8	139.5 ± 10.2

HOMA-IR, homeostasis model assessment of insulin resistance.

Values are mean ± SEM for all groups (chow, *n* = 9/group; HFD + vehicle and L654, *n* = 15/group).

a Indicates *P* value < 0.05 versus chow reference group.

b Indicates *P* value < 0.05 versus HFD + vehicle control.

### L654 reduces liver cholesterol, inflammation, and fibrosis markers in Ldlr^−/−^ mice

**Fig. 2:**
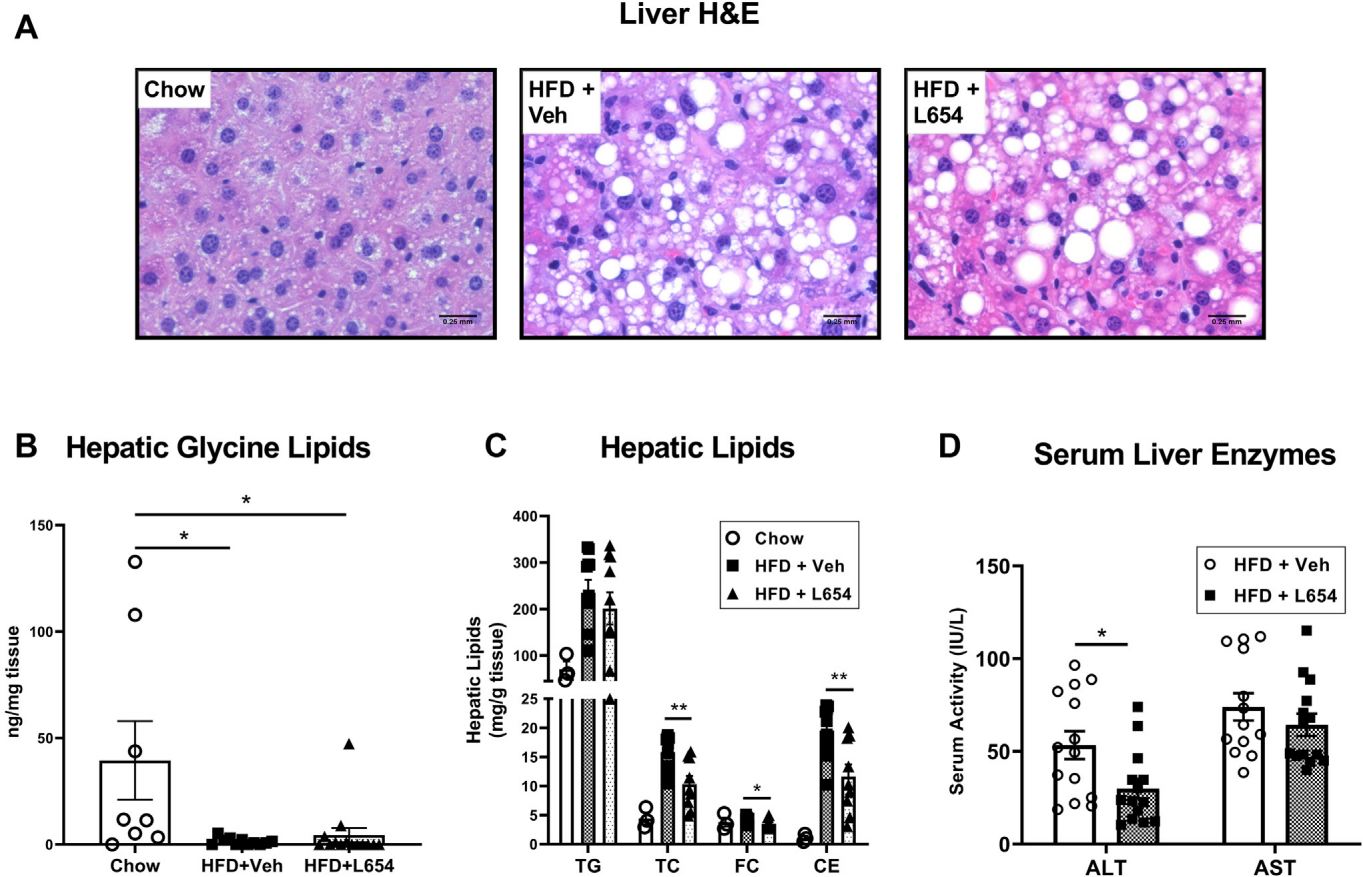
L654-injected *Ldlr*^−/−^ mice have lower hepatic cholesterol and serum ALT. *Ldlr*^−/−^ mice were fed either standard low-fat chow diet (Chow) or Western-type HFD for 14 weeks. Mice fed HFD were intraperitoneally injected with L654 (1 μg) (HFD + L654) or vehicle (HFD + Veh) every 48 h during the final 7 weeks of HFD. Livers were formalin-fixed, sectioned, and H&E stained (A) for visualizing hepatic steatosis (200×). Lipids were extracted from livers using Bligh and Dyer method, and total bacterial glycine lipids (B) were quantified by UPLC-MS/MS (*n* = 8–14, mean ± SEM). Liver lipids were extracted from HFD-fed mice using a modified Folch extraction and measured by enzymatic methods (C), (TG; total cholesterol [TC]; free cholesterol [FC]; cholesteryl ester [CE]) (*n* = 3–10, mean ± SEM). The activity of serum liver enzymes, ALT and aspartate aminotransferase (AST), is shown (D) (*n* = 15, mean ± SEM). Statistical significance determined by two-tailed Student’s *t-*test (∗*P* < 0.05 and ∗∗*P* < 0.01).

Representative images of H&E-stained liver sections are shown in Fig. 2A. Compared with chow-fed mice, both HFD + Veh and HFD + L654 animals had visible microvesicular and macrovesicular steatosis. Hepatic glycine lipids were also lower in both HFD-fed groups compared with those fed a chow diet (Fig. 2B), with 96% and 89% reductions seen in HFD + Veh and HFD + L654, respectively. Mice injected with L654 had significantly reduced hepatic total cholesterol (−35%), free cholesterol (−17%), and cholesteryl esters (−40%) compared with vehicle-injected mice, although there was no difference in hepatic TG content (Fig. 2C). Mice injected with L654 experienced a significant reduction in serum ALT (−43%) compared with the vehicle-injected group (Fig. 2D), indicating L654 attenuated diet-induced liver injury in these animals.

**Fig. 3:**
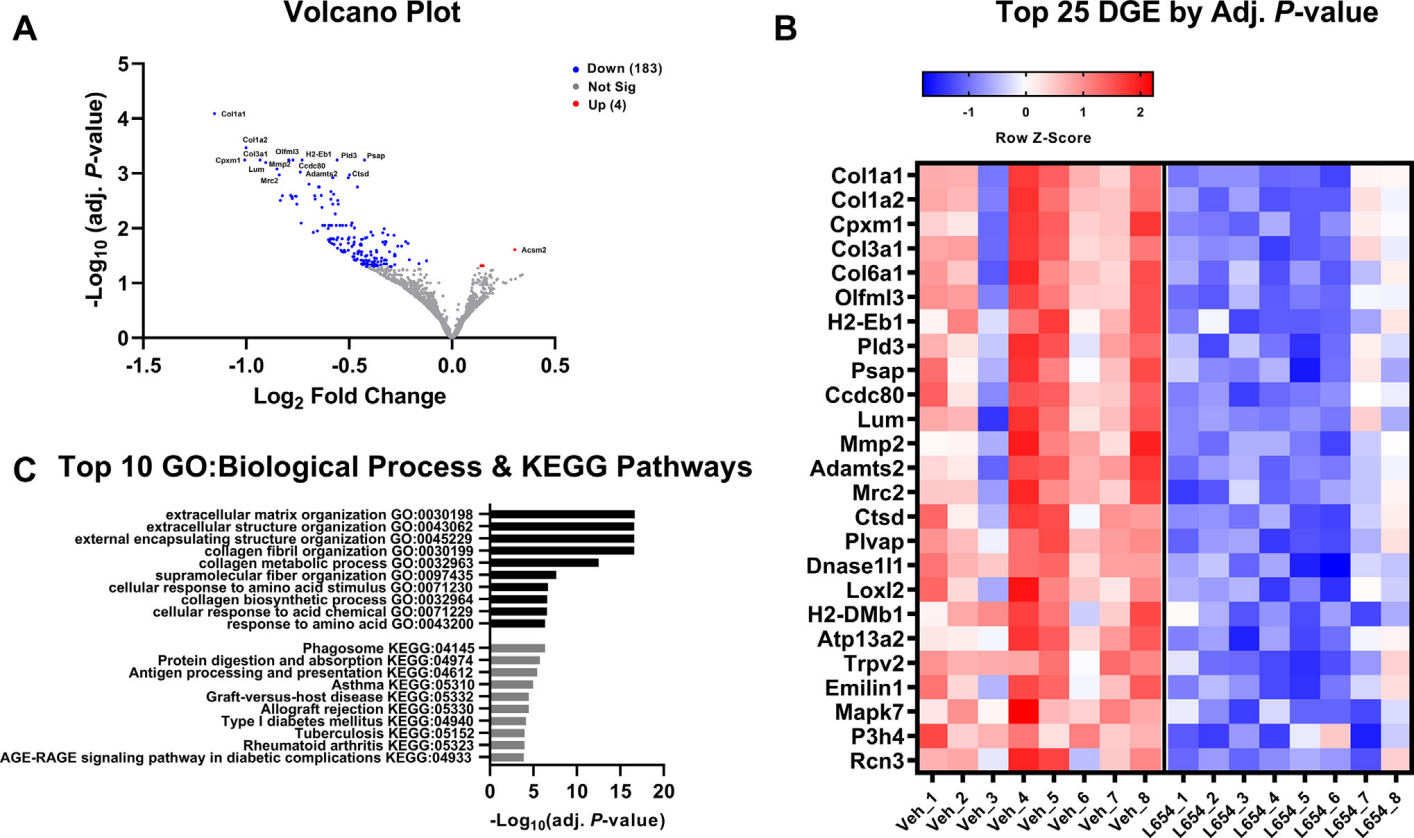
Liver transcriptomics analysis of L654 versus vehicle-treated *Ldlr*^−/−^ mice shows downregulation of extracellular matrix and phagosome-related pathways. RNA-Seq was conducted using snap-frozen liver tissues from Western-type diet-fed *Ldlr*^−/−^ mice injected with vehicle or L654 (1 μg every 48 h) for 7 weeks (*n* = 8 per group). Volcano plot (A) of transcriptome data displaying the pattern of gene expression values in livers of L654-treated mice versus vehicle-treated mice. Significantly differentially expressed genes (adjusted *P* < 0.05) are highlighted in blue (downregulated) and red (upregulated), with the gray data points representing nonsignificant genes. Heatmap (B) of top 25 differentially expressed genes (DEGs). Top 10 Gene Ontology (GO) biological process and KEGG pathways identifying significantly enriched functional categories (C). Log2 fold change, which gives an estimate of the log2 fold change that can be used for ranking genes by the effect size of the treatment, and *P*-adj, the false discovery rate implied by rejecting the null hypothesis for all genes with *P*-adj less than or equal to the given value.

**Fig. 4:**
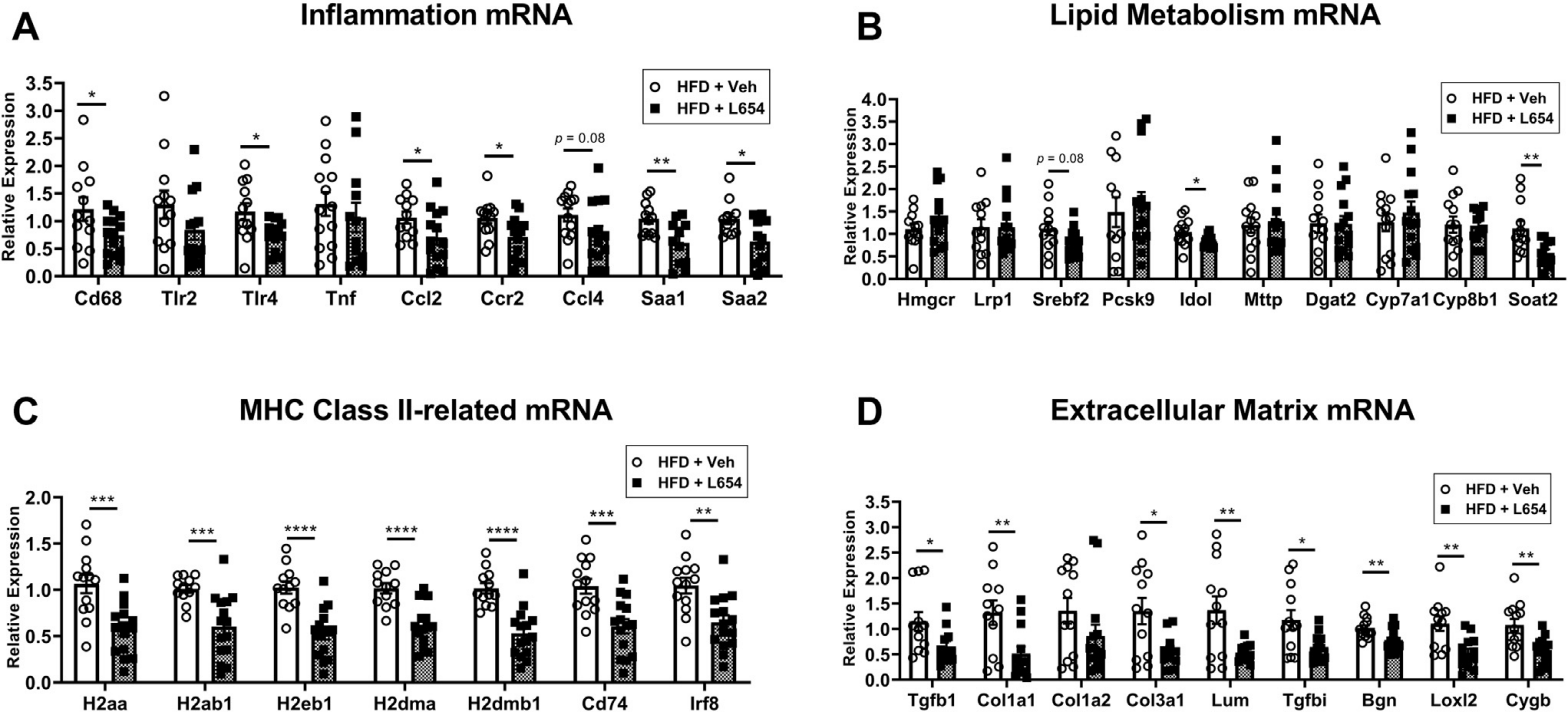
L654 has limited effects on hepatic lipid metabolism mRNA but decreases mRNA of genes involved in inflammation, MHC class II, and extracellular matrix pathways. Hepatic mRNA expression in L654-injected or vehicle-injected Western-type HFD-fed *Ldlr*^−/−^ mice was determined using real-time qRT-PCR and standardized to the geometric mean of Gapdh, β-actin, and Rplp0 reference genes using the 2^−ΔΔCt^ method. Quantification of mRNA expression of genes related to inflammation (A), lipid metabolism (B), MHC class II and antigen processing (C), and extracellular matrix (D) is shown (*n* = 12–14, mean ± SEM). Statistical significance determined by two-tailed Student’s *t-*test (∗*P* < 0.05, ∗∗*P* < 0.01, ∗∗∗*P* < 0.001, and ∗∗∗∗*P* < 0.0001).

To further explore the hepatic effects of L654, we employed RNA-Seq to compare the liver transcriptomes of HFD + Veh and HFD + L654 mice. There were a total of 187 differentially expressed genes after statistical correction (Fig. 3A and supplemental Table S3). One-hundred eighty-three of these genes were downregulated by L654, with modest log2 fold changes (i.e., most 1-fold to 2-fold different). The top 25 differentially expressed genes are shown in Fig. 3B. Many of the pathways affected are implicated in nonalcoholic steatohepatitis and liver fibrosis. The gene set enrichment analysis showed that the changes were highly enriched in collagen-containing extracellular matrix pathways (e.g., *Col1a1*, *Col1a2*, *Col3a1*, *Col6a1*, matrix metalloproteinases being downregulated in HFD + L654 vs. HFD + Veh) (Fig. 3C and supplemental Table S4). There were also changes in phagosome-related pathways (antigen presentation and lysosomal protein catabolism) consistent with a reduced immune response in HFD + L654 livers. Markers known to be enriched in hepatic dendritic cells/macrophages (*Irf8*) and hepatic stellate cells (*Dcn*, *Pdgfrb*, and *Col1a1*) were reduced with L654. These changes in fibrosis and antigen-presenting pathways were confirmed via qPCR (Fig. 4C,D). L654-injected mice had lower hepatic expression (−48% to −38%) of major histocompatibility complex (MHC) class II-related genes (*H2aa*, *H2ab1*, *H2eb1*, *H2dma*, *H2dmb1*, *Cd74*, and *Irf8*) (Fig. 4C). Furthermore, L654-injected mice had lower hepatic expression (−61% to −23%) of extracellular matrix-related genes (*Col1a1*, *Col3a1*, *Tgfb1*, *Lum*, *Tgfbi*, *Bgn*, *Loxl2*, and *Cygb*) (Fig. 4D). Injection with L654 significantly lowered gene expression of several hepatic markers of inflammation (Fig. 4A). Only modest changes were observed in the mRNA of genes involved in lipid metabolism (Fig. 4B). There were no significant differences in the expression of bile acid synthetic genes (*Cyp7a1* and *Cyp8b1*), genes involved in VLDL secretion (*Mttp* and *Dgat2*), or lipoprotein clearance (*Lrp1* and *Pcsk9*). However, some cholesterol responsive genes were altered, including sterol O-acyltransferase 2 (*Soat2*), sterol regulatory element-binding protein 2 (*Srebf2*) (*P* = 0.08), and inducible degrader of LDL receptor (although not active in LDL-receptor null mice), consistent with lower hepatic cholesterol content (Fig. 4B).

**Fig. 5:**
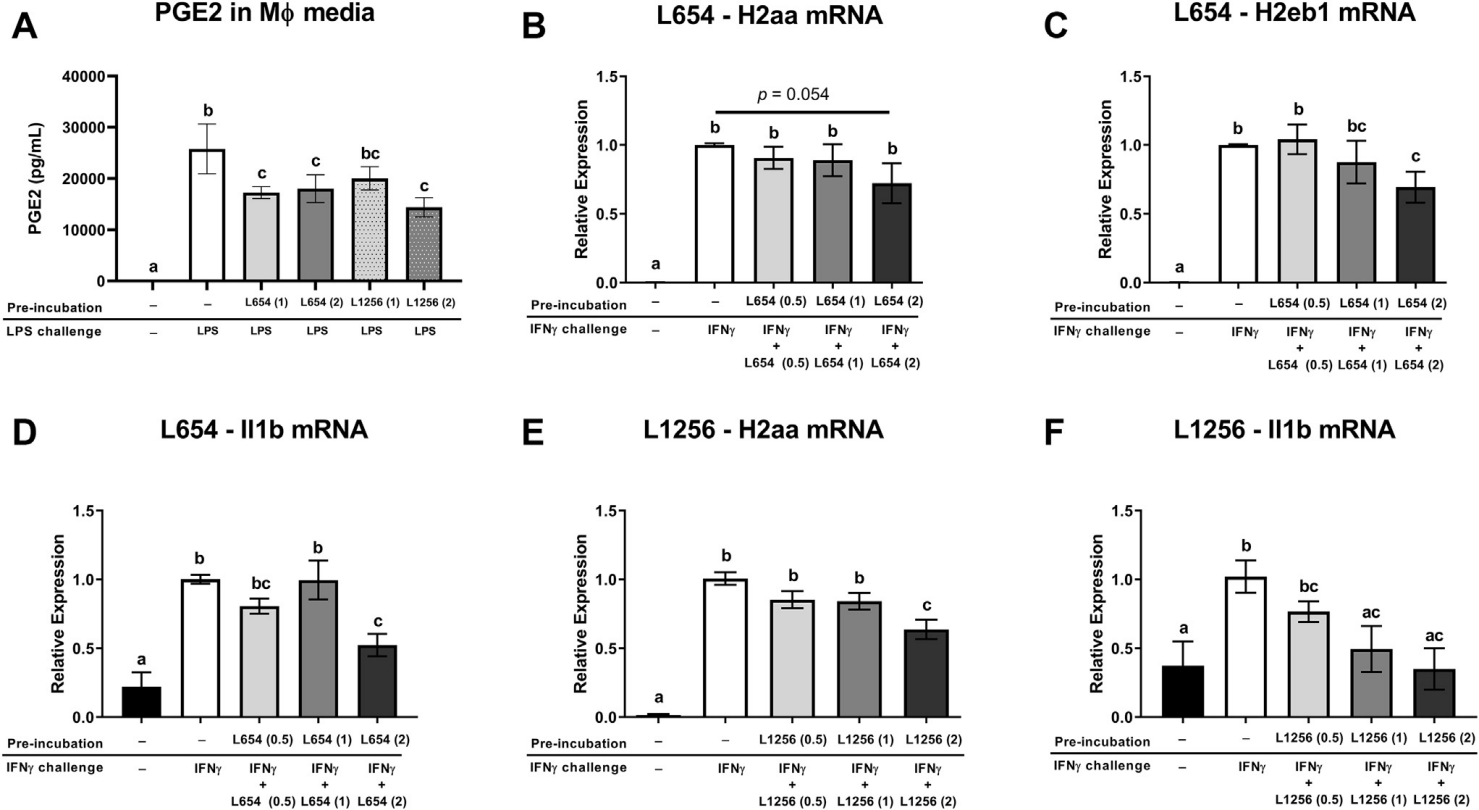
Bacterial glycine lipids reduce inflammatory activation of macrophages treated with LPS or IFNγ. A: RAW264.7 macrophages were incubated with L654 (0.5, 1, or 2 μg/ml), L1256 (0.5, 1, 2 μg/ml), or vehicle control for 24 h and then stimulated with LPS (100 ng/ml) for another 8 h. PGE_2_ in conditioned media was measured by ELISA. B–F: RAW264.7 macrophages were treated with either L654 (0.5, 1, and 2 μg/ml), L1256 (0.5, 1, and 2 μg/ml), or vehicle control for 24 h and then stimulated with recombinant IFNγ (10 ng/ml) for another 24 h in the presence or the absence of the bacterial lipids. mRNA expression of *H2aa* (B, E), *H2eb1* (C), and IL-1β (*Il1b*) (D, F) was measured by real-time qRT-PCR. Values are mean ± SEM, *n* = 4–8 independent experiments. Mean values with different letters indicate differences at *P* < 0.05 using one-way ANOVA with Fisher’s least significant difference for multiple comparisons.

To further elaborate on the physiological effects of glycine lipids in the liver, we tested the in vitro effects of L654 and L1256 in macrophages. Since the gene ontology of RNA-Seq liver data indicated that differentially expressed genes were enriched in phagosome-related pathways and MHC class II-related gene expression, we examined if bacterial glycine lipids modulated macrophage activation and induction of MHC class II genes, *H2aa* and *H2eb1*, in RAW264.7 murine macrophages. We first measured PGE_2_ production and MHC class II gene expression in RAW264.7 macrophages incubated with L654, L1256, LPS or vehicle control for 24 h and then stimulated with LPS for another 8 h. Preincubation for 24 h with L654 (2 μg/ml) or L1256 (2 μg/ml) attenuated LPS-induced PGE_2_ production (Fig. 5A). Preincubation of L654 was also associated with lower *H2aa* (−35% at 2 μg/ml) (*P* = 0.031) and *H2eb1* (−31% at 0.5 μg/ml) (*P* = 0.025) induction by LPS (supplemenal Fig. S6). To further elaborate on the physiological effects of glycine lipids on macrophage activation, we next tested the effects of L654 and L1256 on IFNγ induction of MHC class II gene expression in RAW264.7 macrophages. Cells were treated with L654, L1256, or vehicle control for 24 h and then stimulated with recombinant IFNγ for another 24 h in the presence or the absence of the bacterial lipids. L654 had modest effects on attenuating IFNγ-induced *H2aa* (−28%) (*P* = 0.05), *H2eb1* (−31%) (*P* = 0.046), and IL-1β (*Il1b*) (−48%) (*P* = 0.003) mRNA expression only at the highest concentration tested (2 μg/ml) (Fig. 5B–D). In addition, L1256 showed a significant, yet modest, reduction in *H2aa* (−37%) (*P* < 0.0001) at the highest concentration tested (2 μg/ml) (Fig. 5E). L1256 also reduced IFNγ-induced IL-1β (*Il1b*) mRNA expression at 1 μg/ml (−52%) (*P* = 0.016) and 2 μg/ml concentrations (−67%) (*P* = 0.004) (Fig. 5F).

### L654 attenuates aortic root atherosclerosis progression in Ldlr^−/−^ mice

**Fig. 6:**
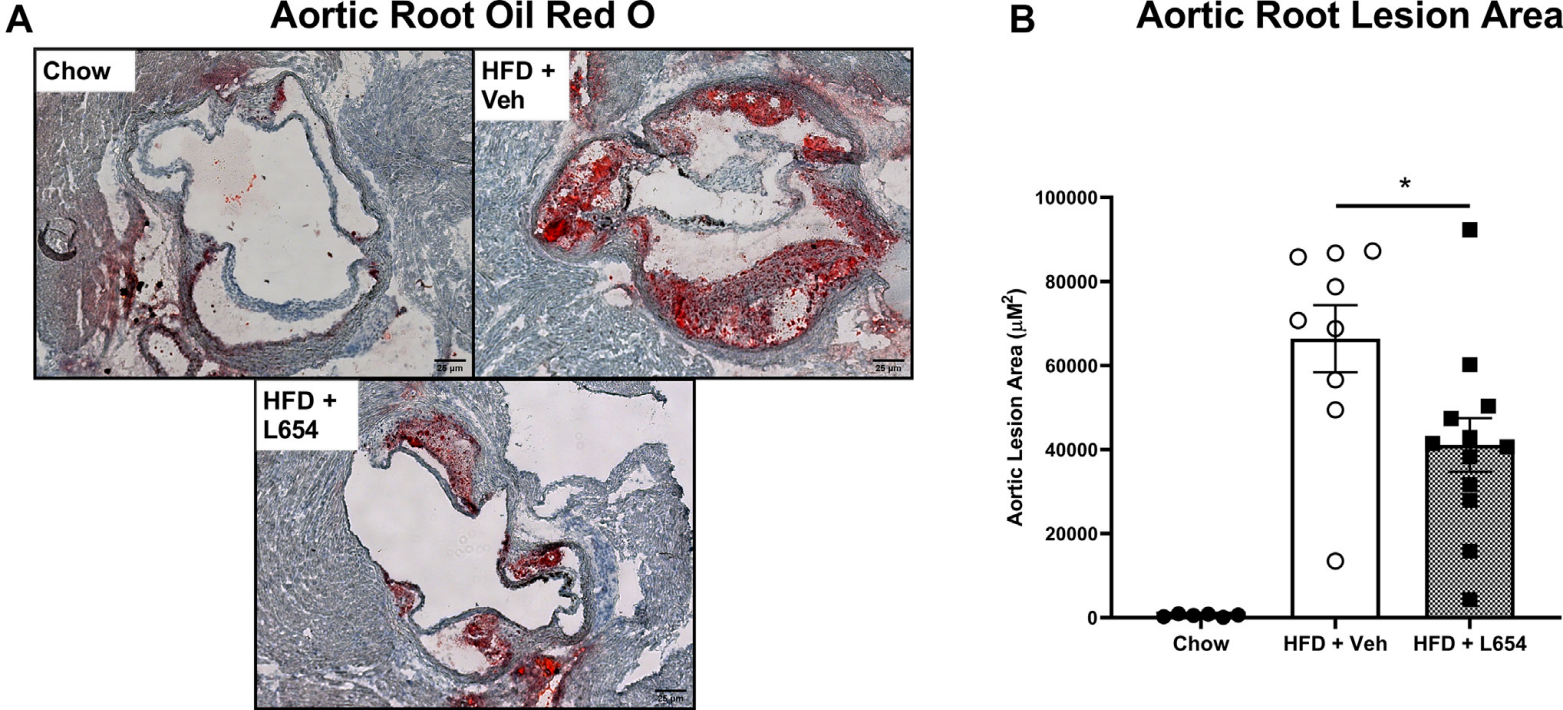
L654 significantly attenuates aortic root lesion development in *Ldlr*^−/−^ mice. Representative images of ORO-stained aortic roots (A) from Western-type HFD-fed *Ldlr*^−/−^ mice injected with vehicle (HFD + Veh) or 1 μg L654 (HFD + L654) every 48 h for 7 weeks. Low-fat chow-fed mice used as a negative control (Chow). Absolute quantification of ORO lesion areas is shown (*n* = 6–12, mean ± SEM). Statistical significance was determined by two-tailed Student’s *t-*test between HFD groups (∗*P* < 0.05). ORO, Oil Red O.

Analysis of aortic roots for plaque revealed that the Western-type HFD was effective in inducing atherosclerosis compared with chow-fed animals (Fig. 6A). Consistent with the observed reductions in serum cholesterol, L654-injected HFD-fed mice had significantly less plaque accumulation (−39%) when compared with the vehicle-injected HFD-fed mice (Fig. 6B). L654-injected mice had a greater concentration of fecal cholesterol (+52%) compared with vehicle-injected mice but had a lower concentration of fecal coprostanol (−49%); consequently, fecal neutral sterol concentrations were not significantly different between the HFD-fed groups (supplemental Table S5).

### L654 has attenuated effects in chow-fed Apoe^−/−^ mice with normal fecal glycine lipid content

**Fig. 7:**
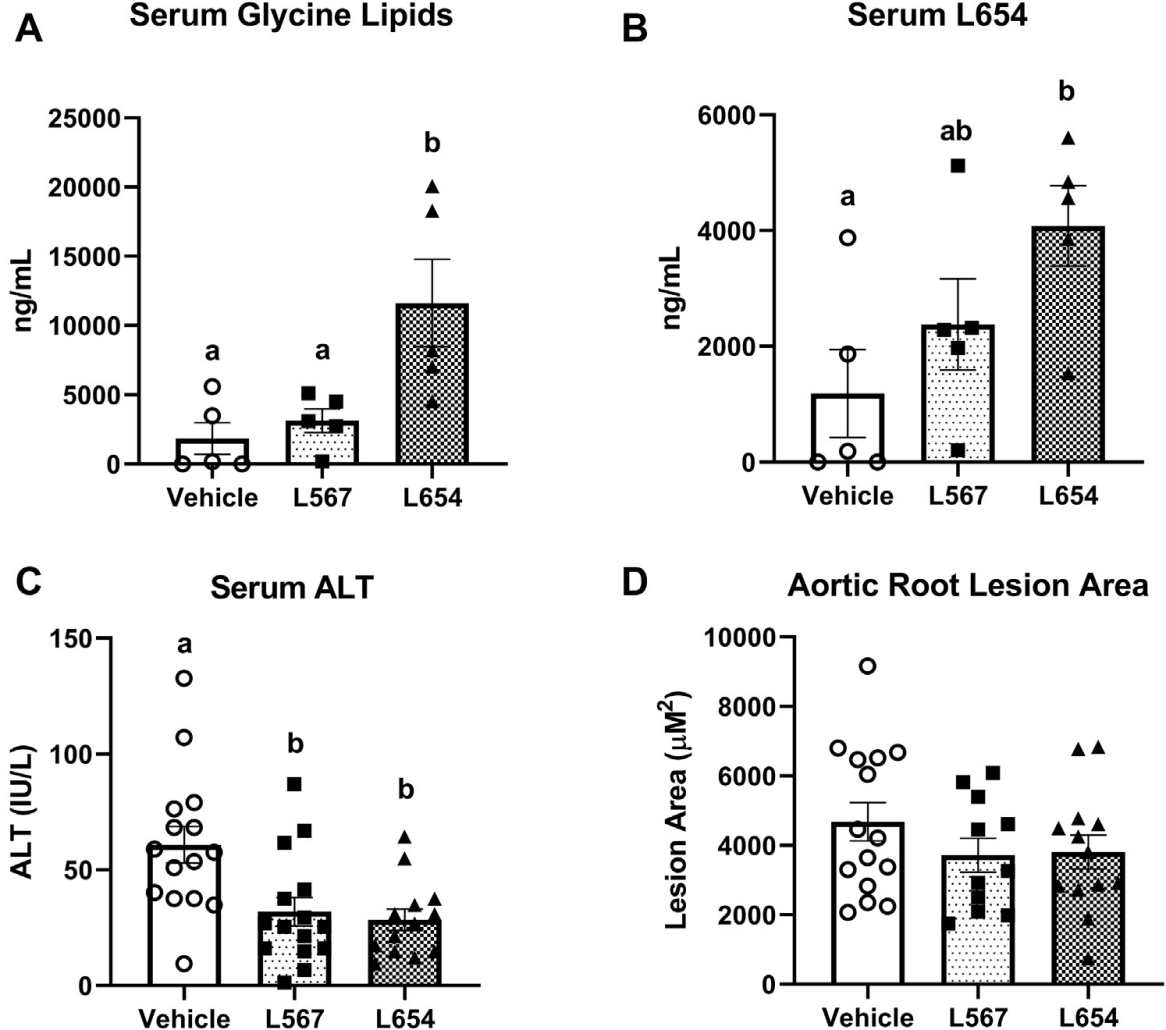
Bacterial glycine lipids reduce liver injury but do not attenuate atherosclerosis in chow-fed *Apoe*^−/−^ mice. About 13-week-old *Apoe*^−/−^ male mice were fed a low-fat chow diet while being intraperitoneally injected every 48 h with either L654 (1 μg), L567 (1 μg), or vehicle control for 7 weeks. Serum glycine lipids (A) and L654 (B) were quantified by UPLC-MS/MS after pooling three individual animals per sample (*n* = 5 pooled, mean ± SEM). The activity of serum ALT is shown (C) (*n* = 15, mean ± SEM). Absolute quantification of ORO lesion areas is shown (D) (*n* = 11–15, mean ± SEM). Mean values with different letters indicate differences at *P* < 0.01 using one-way ANOVA with Fisher’s LSD for multiple comparisons. ORO, Oil Red O.

Since our initial studies of L654 bioactivity relied on feeding a Western-type HFD, which reduces fecal bacterial glycine lipids (including L654), we next investigated the effects of bacterial glycine lipids on atherosclerosis in chow-fed *Apoe*^−/−^ mice. Thirteen-week-old *Apoe*^−/−^ male mice were maintained on a low-fat chow diet while being intraperitoneally injected every 48 h with either L654 (1 μg), L567 (1 μg), or vehicle control for 7 weeks. Chow-fed *Apoe*^−/−^ had comparable levels of total fecal bacterial glycine lipids compared with chow-fed *Ldlr*^−/−^ mice (Table 3). Interestingly, the composition of the bacterial glycine lipids differed between the genotypes, as L1256, the most potent TLR2-activating glycine lipid (13), predominated in *Apoe*^−/−^ fecal samples (∼60% of total), whereas L654 was the major glycine lipid in *Ldlr*^−/−^ samples (∼68% of total). These differences may be explained by the different Bacteroidetes composition found in *Apoe*^−/−^ and *Ldlr*^−/−^ fecal samples (supplemental Fig. S7). We confirmed that the bacterial glycine lipid content of feces is modulated by diet, as feeding *Apoe*^−/−^ mice a Western-type HFD for 2 weeks resulted in a significant reduction (−70%) in total fecal bacterial glycine lipids (Table 4). After 7 weeks of injections, there were no significant differences between the three groups of animals for body and tissue weights (supplemental Table S6) or fecal bacterial glycine lipids (supplemental Table S7). Serum glycine lipids (+259%) (Fig. 7A) were significantly increased with L654 administration but not with L567. This was explained mainly by significant increases in both serum L654 (+244%) (Fig. 7B) and serum L1256 (+246%) (supplemental Fig. S8A). However, like the effect of L654 seen in HFD-fed *Ldlr*^−/−^ mice, both L654 and L567 significantly reduced serum ALT (−53% and 48%, respectively), a marker of liver injury (Fig. 7C). In contrast, neither L654 nor L567 affected serum or hepatic lipids (Table 5). In addition, the antiatherosclerotic effect of L654 seen in HFD-fed *Ldlr*^−/−^ mice was lessened in chow-fed *Apoe*^−/−^ mice, as neither L654 (*P* = 0.23 vs. vehicle) nor L567 (*P* = 0.20 vs. vehicle) significantly affected aortic lesion area (Fig. 7D). Bacterial glycine lipids were detected in aortic tissue samples, but no significant differences were observed between groups for any of the lipid species (supplemental Fig. S8B–D).

**Table 3:** Fecal bacterial glycine lipids of chow-fed *Apoe*^−/−^ mice and *Ldlr*^−/−^ mice

Variable	*Apoe*^−/−^	*Ldlr*^−/−^
Total glycine lipids (ng/g)	15,911 ± 1,141	13,836 ± 4,169
L1256 (ng/g)	9,594 ± 620^a^	676 ± 93
L654 (ng/g)	728 ± 339^b^	9,402 ± 3,737
L567 (ng/g)	1,437 ± 179^c^	2,824 ± 512
L430 (ng/g)	2,974 ± 1,013	671 ± 249
L342 (ng/g)	1,178 ± 187^c^	263 ± 89

Values are mean ± SEM for all groups (*n* = 3–12/group).

a Indicates *P* value < 0.0001 versus *Ldlr*^−/−^ on chow.

b Indicates *P* value < 0.001.

c Indicates *P* value < 0.05.

**Table 4:** Fecal bacterial glycine lipids of *Apoe*^−/−^ mice on chow and HFDs for 2 weeks

Variable	Chow-*Apoe*^−/−^	HFD-*Apoe*^−/−^
Total glycine lipids (ng/g)	30,333 ± 2,355	8,985 ± 312^a^
L1256 (ng/g)	7,213 ± 654	4,011 ± 761
L654 (ng/g)	935 ± 631	ND
L567 (ng/g)	1,001 ± 214	661 ± 214
L430 (ng/g)	20,405 ± 2,231	3,379 ± 1,201^b^
L342 (ng/g)	780 ± 63	926 ± 374

ND, not detected.

Values are mean ± SEM for all groups (*n* = 4/group).

a Indicates *P* value < 0.01.

b Indicates *P* value < 0.001 versus chow-*Apoe*^−/−^.

**Table 5:** Serum and liver biochemical analysis of *Apoe*^−/−^ mice

Variable	Vehicle	L567	L654
Total cholesterol (mg/dl)	472.0 ± 27.3	436.4 ± 25.9	491.9 ± 13.5
HDL-C (mg/dl)	31.3 ± 2.4	26.2 ± 9.2	30.2 ± 3.1
Non-HDL-C (mg/dl)	440.7 ± 27.6	410.3 ± 25.2	461.7 ± 13.8
TGs (mg/dl)	113.6 ± 8.5	96.0 ± 8.7	100.6 ± 6.7
Liver cholesterol (mg/g)	9.38 ± 0.53	9.35 ± 0.50	8.94 ± 0.66
Liver TGs (mg/g)	71.54 ± 9.98	54.51 ± 6.87	62.11 ± 4.83

Values are mean ± SEM for all groups (*n* = 15/group).

## Discussion

The recently identified bacterial glycine lipids are putative virulence factors produced by microbes of the phylum Bacteroidetes. Despite evidence suggesting that L654 and other glycine lipids activate TLR2 signaling, we report here protective effects against hepatic inflammation and atherosclerosis with chronic exposure, particularly in HFD conditions where fecal and serum glycine lipids are reduced. We found that 7-week intraperitoneal administration of L654 (1 μg every 48 h) to HFD-fed *Ldlr*^−/−^ mice lowered cholesterol, attenuated atherosclerosis progression, and decreased markers of liver injury compared with vehicle control-injected mice. L654 reduced liver inflammatory and extracellular matrix gene expression, which may be related to an attenuation of macrophage activation in the liver, as demonstrated by downregulation of MHC class II gene expression as shown via RNA-Seq, qRT-PCR, and confirmed in macrophage cell experiments. We also report that, compared with chow-fed mice, L654 and other bacterial glycine lipids were markedly reduced alongside changes in Bacteroidetes relative abundance in the feces, serum, and liver tissues of HFD-fed mice, with reductions in feces seen in as little as 2 weeks after HFD feeding. Thus, it appears that HFD-fed obese mice are exposed to fewer gut Bacteroidetes-derived glycine lipids than chow-fed lean animals. The bioactivities of glycine lipids were next tested in chow-fed *Apoe*^−/−^ mice, which displayed much higher fecal glycine lipids compared with HFD-fed *Ldlr*^−/−^ mice. Administration of L654 and L567 for 7 weeks to chow-fed *Apoe*^−/−^ mice reduced the liver injury marker, ALT, but other effects seen in *Ldlr*^−/−^ were not observed in this model. Since there was no HFD or obesity to modify gut microbiota-derived glycine lipids in the chow-fed *Apoe*^−/−^ model, our results suggest that in conditions where gut microbiome-derived TLR2 signals are lost (e.g., HFD-induced obesity), hyperlipidemia and changes in liver immune response may be exacerbated—although, the attenuated effects observed in chow-fed *Apoe*^−/−^ mice may also be explained by genotype differences between the models. Regardless, our findings suggest that chronic exposure to microbiota-related glycine lipids, such as L654, may dampen inflammation-related pathways via TLR2 and prevent atherosclerosis progression and liver injury. Notably, the glycine lipids examined in this study do not appear to be as potent by mass at activating TLR2 as the commonly used synthetic ligands Pam2Cys and Pam3Cys (12). This may explain why other studies administering Pam3Cys reported an exacerbation of atherosclerosis in *Ldlr*^−/−^ mice, despite also lowering serum cholesterol in the animals (22).

As data regarding the bioactivities of bacterial glycine lipids are limited, it is helpful to compare their effects to other TLR2 agonists in the context of lipid metabolism and atherosclerosis. Interestingly, Mullick *et al.* (22) demonstrated that weekly injection of 25 μg Pam3Cys in female *Ldlr*^−/−^ mice on an HFD for 12 weeks significantly lowered measures of total serum cholesterol. Consistent with their findings, we are reporting that administration of 1 μg of L654, a much weaker TLR2 agonist, every 48 h for 7 weeks also reduced serum cholesterol in *Ldlr*^−/−^ mice. The exact mechanism of the effect of L654s on lipid metabolism is unclear; however, lower hepatic gene expression of SREBP2 (*P* = 0.08) and sterol O-acyltransferase 2 may partly explain these findings in the liver and serum. The lower inducible degrader of LDL receptor mRNA expression seen in livers of L654-injected mice is potentially because of lower hepatic cholesterol and would not influence serum cholesterol in *Ldlr*^−/−^ mice. However, changes in hepatic and serum cholesterol pools could not be explained by differences in fecal neutral sterol analysis. *Ldlr*^−/−^ mice injected with L654 had higher fecal cholesterol content than mice administered the vehicle control; however, the vehicle-treated group had greater fecal cholesterol metabolites (particularly coprostanol), so the total fecal neutral sterols were not significantly different between the HFD groups. With the reduction in serum cholesterol in *Ldlr*^−/−^ mice, it is reasonable to expect a protection against atherosclerosis. Indeed, we found that aortic root lesions were significantly reduced by L654 in *Ldlr*^−/−^ mice. However, Mullick *et al.* (22) found an increase in atherosclerotic plaque with injection of Pam3Cys, despite a similar reduction in serum cholesterol as we observed in our study. These disparate findings may be explained by the variable potency of the respective TLR2 agonists examined. In addition, L654 has been shown to engage the heterodimer receptor TLR2/TLR6, whereas Pam3Cys activates TLR2/TLR1 (12, 13). Diacylated lipopeptides have been shown to have immunosuppressive effects via TLR2/TLR6, which is not seen with triacylated lipopeptides that signal through TLR2/TLR1 (23). Future studies should explore the mechanism underlying the effects of TLR2 agonists on cholesterol metabolism reported in the literature.

As the intraperitoneal injection of substances can lead to absorption via the portal vein, it was unsurprising that a major effect of glycine lipids was seen in the liver. Because activation of TLRs is widely associated with upregulation of inflammatory pathways and 1 μg L654 intraperitoneal injection in mice was previously shown to acutely elevate serum CCL2 (11), we initially expected L654 would increase markers of inflammation. Surprisingly, we found the opposite in the liver. Furthermore, after 7 weeks of injection, L654 had no effect on serum inflammatory markers (i.e., SAA, CCL2, or IL-1β) compared with vehicle-injected mice on the same HFD. However, it is important to note that Clark *et al.* (11) implemented a single injection of L654 to mice, whereas the current study employed chronic administration over 7 weeks. In addition, this injection occurred in wild-type C57BL/6 mice fed chow, a diet condition that likely resulted in higher exposure to glycine lipids present in the colon/feces than seen in the HFD-fed mice of the present study. Another study reported a dose-dependent increase in circulating SAA in the 24 h after injection of 25 and 50 μg Pam3Cys in *Ldlr*^−/−^ mice (22). Thus, perhaps acute exposure to bacterial glycine lipids results in a transient upregulation of systemic circulation, yet long-term exposure acts differently. We observed a broad reduction of MHC class II gene-related expression in the liver of L654-treated mice, which may be interpreted as a reduction in macrophage activation. This notion was confirmed through in vitro studies in macrophages incubated with L654 or L1256 for 24 h, which then displayed reduced inflammatory activation in the presence of LPS and IFNγ. Although relatively modest attenuations were observed, our findings are consistent with previously reported inhibitory effects of macrophage TLR2 activation on IFNγ-induced MHC-class II expression and processing (24, 25, 26, 27) as well as effects of L654 on TLR2 tolerance in bone marrow-derived macrophages (28). Kubinak *et al.* (29) suggested that low-grade activation of TLRs via commensal bacteria can elicit an adaptive response, that initially will activate inflammatory pathways, but eventually result in host tolerance to the insult. Persistent exposure to TLR2 ligands could make macrophages refractory to IFNγ and decrease their ability to process and present antigens from bacteria via MHC class II. This would be important in dampening a chronic inflammatory response but could be used as a strategy by pathogens to evade the immune system (30). In line with this, short-term exposure (<6 h) to the *Mycobacterium tuberculosis* 27 kDa lipoprotein, LprG, stimulated TLR2-dependent TNF-α production in macrophages; however, long-term exposure (>16 h) of macrophages to LprG markedly inhibited MHC class II antigen processing via TLR2 (31). Synthetic TLR2 agonists (24, 26) and lipoproteins derived from *M. tuberculosis* (25, 27, 31, 32, 33, 34) have previously been demonstrated to inhibit IFNγ-induced expression of MHC class II genes in macrophages via TLR2 signaling.

The liver is normally biased toward features of immunological tolerance rather than immunity. Under steady-state conditions, the normal liver consists of “tolerogenic” antigen-presenting cells, with low expression of MHC class II (35). The liver typically has very low numbers of dendritic cells, whereas resident Kupffer cells, the major macrophages of the liver, display lower MHC class II expression than dendritic cells (36). These features of the liver are important to resist stimulation because of exposure of gut bacterial products arriving via portal venous blood (35). Under steady-state conditions, Kupffer cells take up significant amounts of bacterial products without inducing a proinflammatory response (37). However, under proinflammatory conditions or a high lipid load, there is a break in tolerance where MHC class II expression in the liver is increased (37, 38). This appears to be due to both increases from resident antigen-presenting cells as well as recruitment of circulating monocytes leading to accumulation of proinflammatory macrophages in the tissue (38, 39, 40). Kupffer cells and recruited inflammatory macrophages can also contribute to the activation of hepatic stellate cells and the expression of fibrogenic gene expression in the early stages of nonalcoholic steatohepatitis (40). We observed a clear downregulation of extracellular matrix and collagen gene expression in livers of L654-injected *Ldlr*^−/−^ mice. Previously, L654 was shown to lower osteoblast collagen gene transactivation via TLR2 (15). Wang *et al.* (15) cultured *Tlr2*^−/−^ and wild-type osteoblasts expressing the type I collagen (*Col1a1*) promoter-driven transgene, Col2.3GFP, and then treated the cells with bacterial glycine lipids (L430 and L654). Both L654 and L430 significantly reduced Col2.3GFP expression in wild-type osteoblasts, but these effects were ablated in *Tlr2*^−/−^ cells (15). Despite previous findings, the effects of L654 on fibrogenic gene expression in the current study were likely because of macrophage modulation, as the expression of TLR2 in hepatic stellate cells is very low (unpublished findings).

L654 has previously been implicated in tolerance immunity for the autoimmune disorder, multiple sclerosis (28). Clark *et al.* (28) found that induction of TLR2 tolerance via L654 administration protected against the adoptive transfer of murine experimental autoimmune encephalomyelitis, used as a mouse model of human multiple sclerosis. Low level (5 μg) of administration of L654 for 5 days significantly reduced circulating TNF-α and dramatically reduced disease severity in the experimental autoimmune encephalomyelitis model. These results appeared to be dependent on the interaction of L654 with TLR2, since the well-established agonist of TLR2, Pam2Cys, showed similar effects (28). As the same group also found lower L654 concentrations in serum of patients with multiple sclerosis (41), it was suggested that steady-state exposure to TLR2-tolerizing bacterial products originating from the gut microbiome helps to protect against autoimmune diseases (42). While higher levels of commensal oral and gut bacterial glycine lipids may bear out to be protective against autoimmune and chronic inflammatory diseases, the immune-tolerizing effects of glycine lipids derived from pathogens may allow them to evade immune recognition, which is seen with *P. gingivalis* in periodontal disease (43). Furthermore, diacylated lipopeptides are known to induce acute inflammation followed by immune suppression, such as that seen with *Staphylococcus aureus* during atopic dermatitis (23).

As expected, the feeding of HFD to mice resulted in significant changes in gut microbiota diversity measures and taxa composition when compared with chow-fed animals. Obesity and dietary intake of fat are often associated with reductions in the relative abundance of the Bacteroidetes phylum in the feces of animals and humans (5, 7). The fecal content of glycine lipids of *Ldlr*^−/−^ mice was markedly reduced by 14 weeks of HFD feeding, mirrored Bacteroidetes relative abundance, and was inversely related with body weight. We confirmed the HFD-driven modulation of fecal glycine lipid levels in *Apoe*^−/−^ mice in as little as 2 weeks, prior to any onset of obesity. This modulation is expected to result in a dramatic difference in host exposure to the bacterial glycine lipids present in the gut microbiome. In line with this, we observed significant reductions in serum and hepatic content of glycine lipids in HFD-fed *Ldlr*^−/−^ mice. The chronic administration of L654 partially corrected serum levels; however, accumulation of glycine lipids in liver remained low. Accounting for differences in stool output between HFD and chow-fed animals, it could be estimated that about 15–30 μg of glycine lipids was in the daily stool output of chow-fed mice from our studies, whereas only about 1–2 μg of glycine lipids was in the stool of HFD-fed animals. Bacteroidetes are primarily polysaccharide degraders (44), and thus, it was likely the high fat/low polysaccharide content of the Western-type HFD that drove the changes in fecal glycine lipids in the current study. However, more research is needed to elucidate further effects of dietary components on glycine lipid content and composition of the gut microbiome.

In conclusion, the current investigation shows that Bacteroidetes-derived fecal glycine lipids are significantly reduced with Western-type HFD, and that chronic intraperitoneal exposure to the microbiota-derived serine-glycine lipodipeptide, L654, may prevent atherosclerosis progression and liver injury via hypolipidemic and anti-inflammatory effects. The lower MHC class II gene expression seen in L654-treated mice suggests that liver tolerance was partially maintained during the inflammatory insult of Western-type HFD feeding. A breakdown of tolerance, because of a change in the gut microbiome or other aspects of HFD or obesity, may induce an inappropriate immune response, resulting in acute and chronic inflammatory liver diseases. Therefore, conditions where gut microbiome-derived glycine lipids are lost, such as HFD-induced obesity, may exacerbate development of atherosclerosis and liver injury, while correction of such depletion may attenuate these disorders. More research is warranted to understand the role of bacterial glycine lipids and other TLR2 agonists on host lipid metabolism.

## Data availability

The datasets used and/or analyzed during the current study are available on request from the corresponding author.

## Supplemental data

This article contains supplemental data.

## Conflict of interest

The authors declare that they have no conflicts of interest with the contents of this article.
