# Supplementary material for: Gut microbiome-derived glycine lipids are diet-dependent modulators of hepatic injury and atherosclerosis
Source: J Lipid Res. 2022 Mar 10;63(4):100192. doi: 10.1016/j.jlr.2022.100192 (PMC9020096; doi:10.1016/j.jlr.2022.100192)
Supplement: Supplemental Figures and Tables [file mmc1.docx]

**Supplemental Information:**

**Gut Microbiome-Derived Glycine Lipids Are Diet-Dependent Modulators of Hepatic Injury and Atherosclerosis**

Courtney L. Millar^1,2^, Liya Anto^1^, Chelsea Garcia^1^, Mi-Bo Kim^1^, Anisha Jain^1^, Anthony A. Provatas^3^, Robert B. Clark^4,5^, Ji-Young Lee^1^, Frank C. Nichols^6^, and Christopher N. Blesso^1^

^1^Department of Nutritional Sciences, University of Connecticut, Storrs, CT, USA

^2^The Marcus Institute for Aging Research, Harvard Medical School, Boston, MA, USA

^3^Center for Environmental Sciences and Engineering, University of Connecticut, Storrs, CT, USA

^4^Department of Immunology, UConn Health, Farmington, CT, USA

^5^Department of Medicine, UConn Health, Farmington, CT, USA

^6^Departmentof Periodontology, UConn Health, Farmington, CT, USA

**
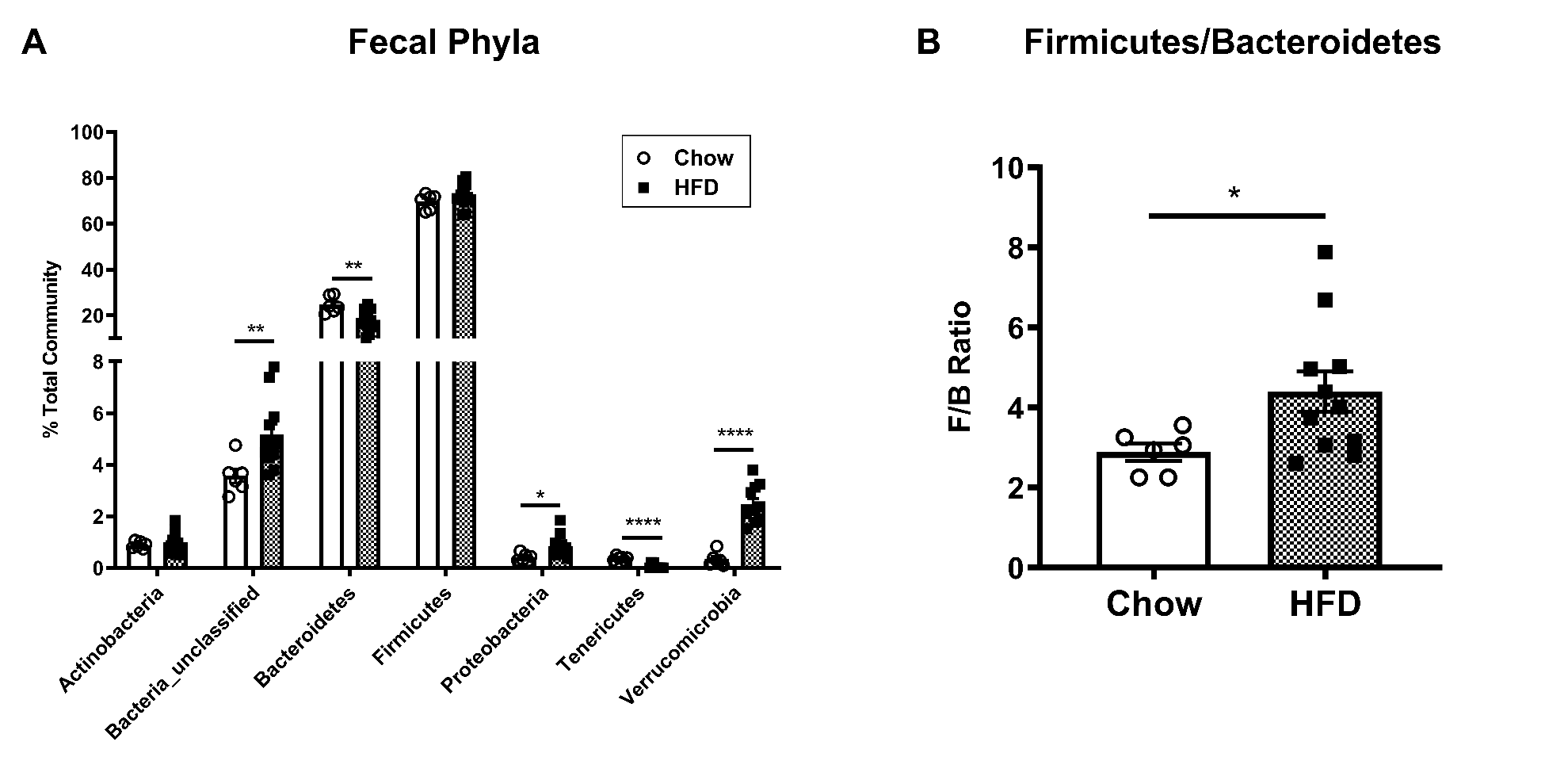
**

**Fig. S1.** **Western-type high-fat diet modulates the phyla abundances of cecal feces microbiota.** (A, B)**:** Cecal feces were aseptically collected for characterization of 16S V4 region. Relative abundances at phyla (A) and the Firmicutes/Bacteroidetes ratio (B). Values are mean ± SEM (*n* = 6-11). Statistical significance determined by two-tailed Student’s *t*-test (**P* < 0.05, ***P* < 0.01, *****P* < 0.0001).

**
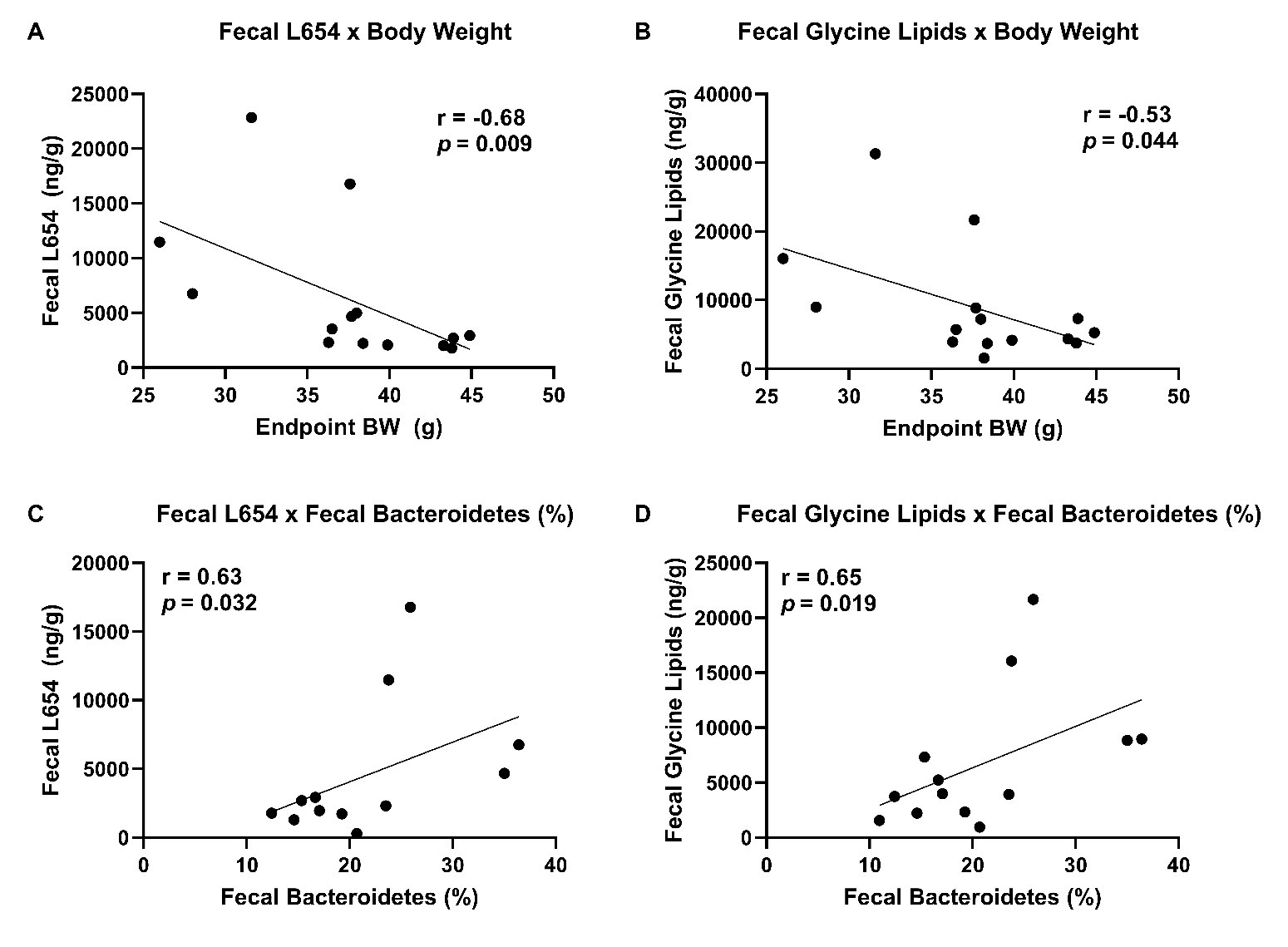
**

**Fig. S2.** **Fecal L654 is negatively associated with body weight and positively associated with fecal Bacteroidetes.** Bivariate Spearman correlations of fecal L654 concentrations with body weight (A) and fecal Bacteroidetes (C). Spearman correlations of fecal total fecal glycine lipids with body weight (B) and fecal Bacteroidetes (D).


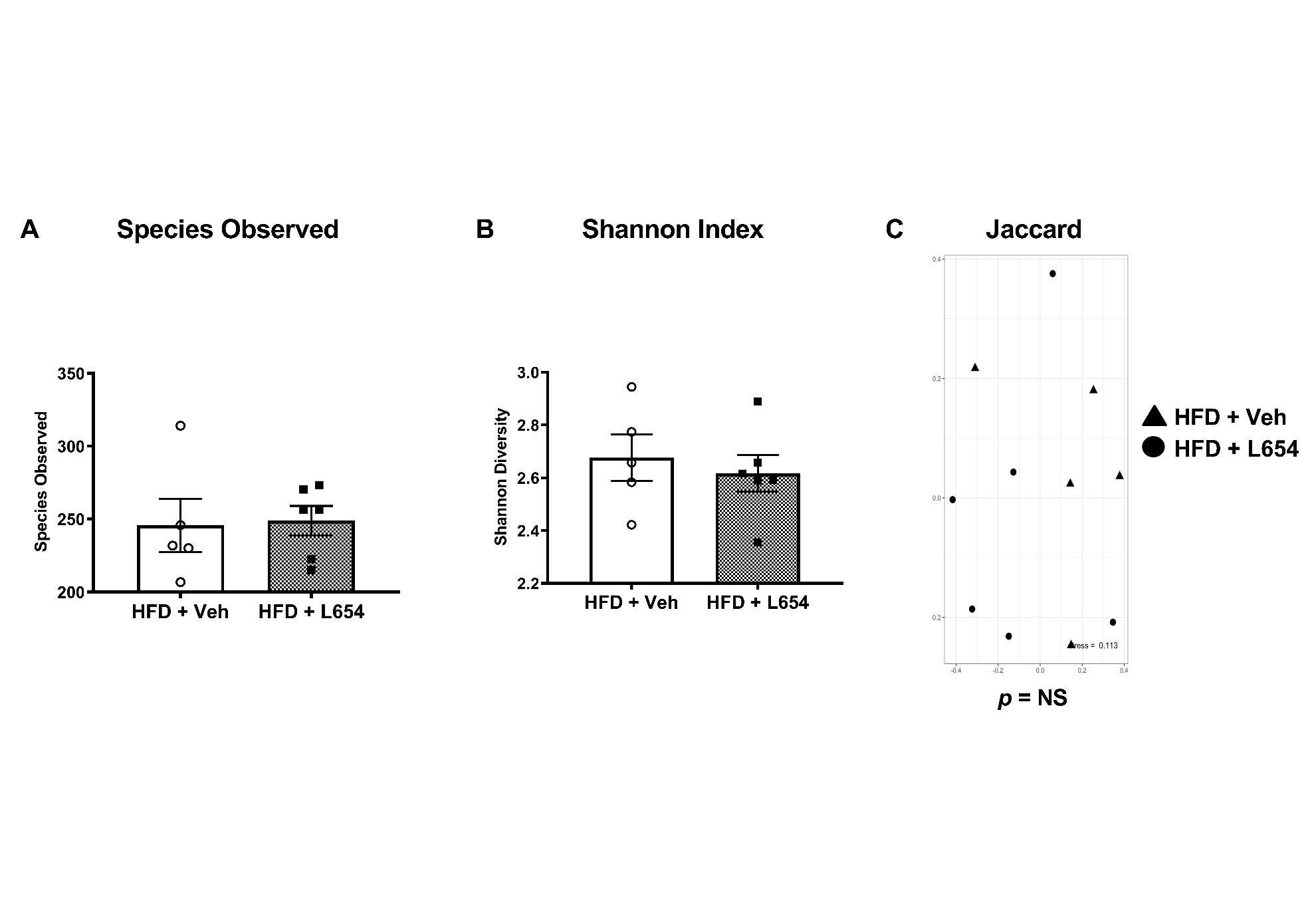


**Fig. S3.** **L654 injections did not alter fecal microbiota diversity in Western-type HFD-fed *Ldlr*^-/-^ mice.** Cecal feces were aseptically collected for characterization of 16S V4 region. Measures of fecal microbiota alpha diversity [species observed (A) and Shannon index (B)], and beta diversity [Jaccard index (C)], of Western-type HFD-fed *Ldlr*^-/-^ mice injected with either L654 (HFD + L654) or vehicle control (HFD + Veh). Data were analyzed via NMDS (Jaccard) or two-tailed Student’s *t*-test (alpha diversity) with *P*<0.05 considered significant. Values are mean ± SEM (*n* = 5-6).


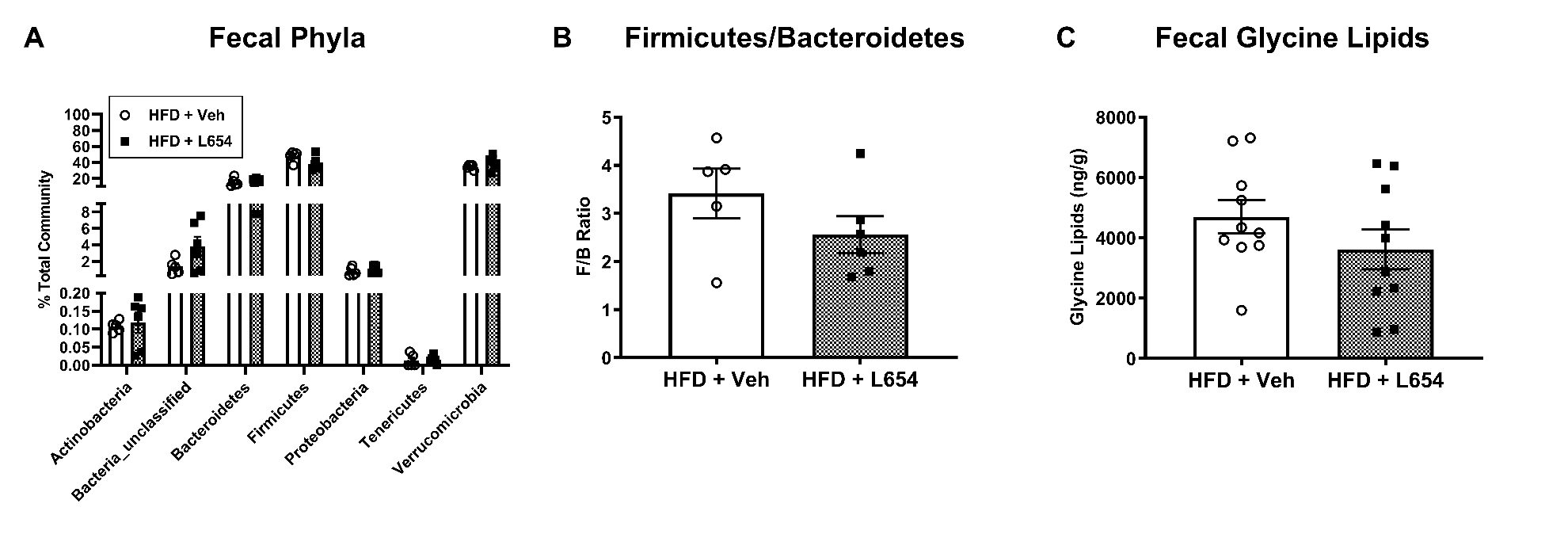


**Fig. S4.** **L654 injections did not alter fecal Bacteroidetes or fecal glycine lipids in Western-type HFD-fed *Ldlr*^-/-^ mice.** (A, B)**:** Cecal feces were aseptically collected for characterization of 16S V4 region. Relative abundances at phyla (A) and the Firmicutes/Bacteroidetes ratio (B) (*n* = 6, mean ± SEM). Lipids were extracted from feces and total bacterial glycine lipids (C) were quantified by UPLC-MS/MS (*n* = 10, mean ± SEM). Data were analyzed by two-tailed Student’s *t*-test with *P*<0.05 considered significant.

**
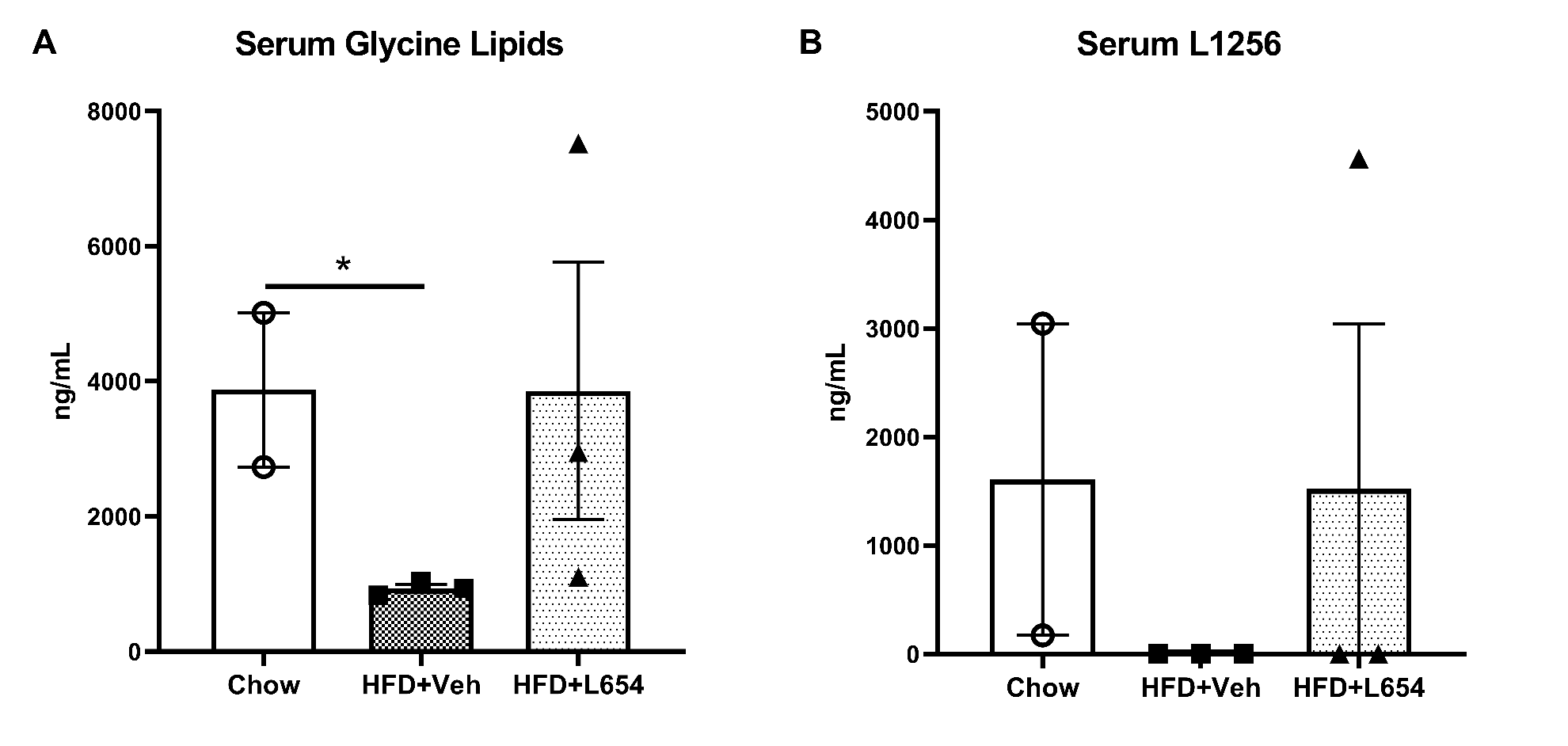
**

**Fig. S5.** **Serum glycine lipids are reduced in Western-type HFD-fed *Ldlr*^-/-^ mice.** Serum glycine lipids were quantified by UPLC-MS/MS after pooling 3 individual animals per sample (*n* = 2-3 pooled, mean ± SEM). Statistical significance determined by two-tailed Student’s *t*-test (**P* < 0.05).

**
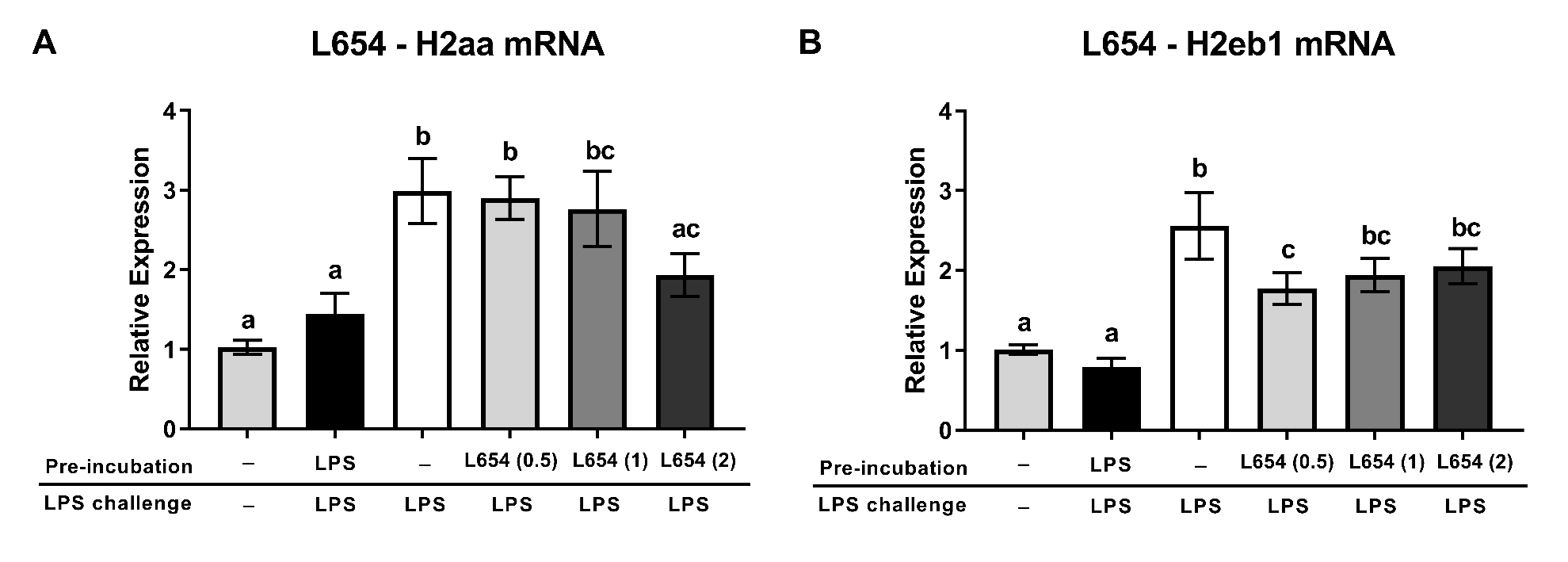
**

**Fig. S6.** **L654 reduces MHC class II gene expression in macrophages treated with LPS.** RAW264.7 macrophages were incubated with L654 (0.5, 1, or 2 µg/mL), LPS (100 ng/mL) or vehicle control for 24 h and then stimulated with LPS (100 ng/mL) for another 8 h. mRNA expression of *H2aa* (A) and *H2eb1* (B) were measured by real-time qRT-PCR. Values are mean ± SEM, *n* = 4-8 independent experiments. Mean values with different letters indicate differences at *P* < 0.05 using one-way ANOVA with Fisher’s LSD for multiple comparisons.

**
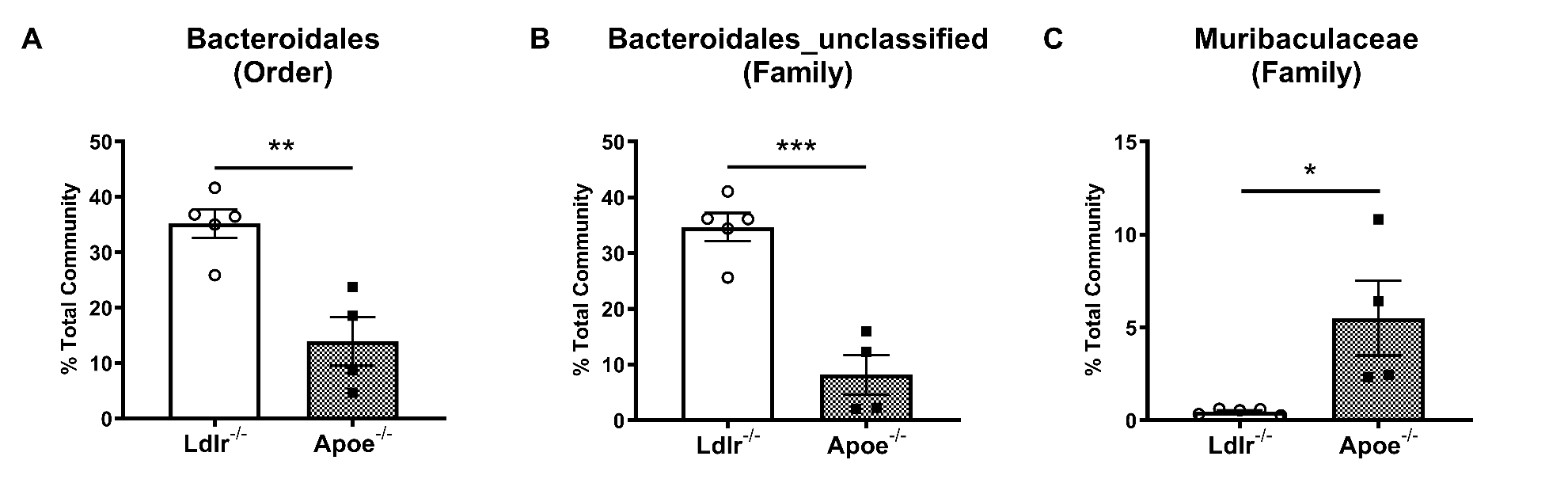
**

**Fig. S7. Compositional differences in** **fecal** **Bacteroidetes from chow-fed *Apoe*^-/-^ and *Ldlr*^-/-^ mice.** Cecal feces were aseptically collected for characterization of 16S V4 region. Relative abundances at phyla (A) and the Firmicutes/Bacteroidetes ratio (B) (*n* = 4-5, mean ± SEM). Data were analyzed by two-tailed Student’s *t*-test (**P* < 0.05, ***P* < 0.01, ****P* < 0.001).

**
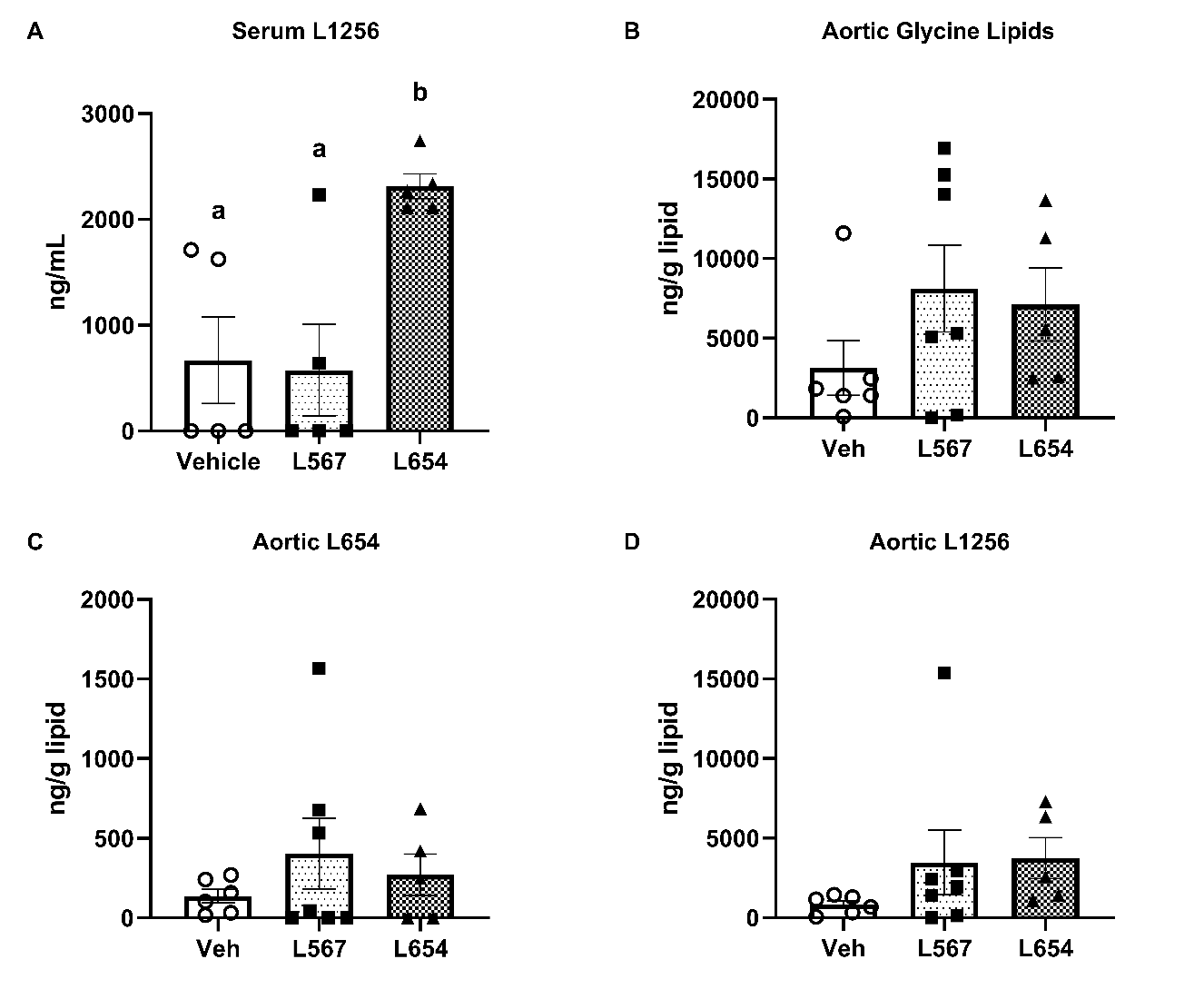
Fig. S8.** **L654 injections increase serum L1256 but do not affect aortic glycine lipids in *Apoe*^-/-^ mice.** Serum L1256 (A) was quantified by UPLC-MS/MS after pooling 3 individual animals per sample (*n* = 5 pooled, mean ± SEM). Lipids were extracted from abdominal aorta using Bligh and Dyer method and total aortic bacterial glycine lipids (B), aortic L654 (C), and aortic L1256 (D) were quantified by UPLC-MS/MS (*n* = 5-7, mean ± SEM).

**Table S1. Composition of Western-type high-fat diet**

| **Diet Component** | **(g/kg)** |
| --- | --- |
| Casein | 195 |
| L-Cystine | 3 |
| Sucrose | 340 |
| Corn Starch | 55.5 |
| Maltodextrin | 60 |
| Anhydrous Milkfat | 210 |
| Soybean Oil | 20 |
| Cellulose | 50 |
| Mineral Mix, AIN-93-MX | 43 |
| Vitamin Mix, AIM-93-VX | 19 |
| Choline Bitartrate | 3 |
| TBHQ | 0.04 |
| Cholesterol | 1.5* |

*Milk fat contributed 0.05% (w/w) cholesterol, resulting in 0.2% total cholesterol (w/w) in diets

**Table S2. Primer List for qRT-PCR**

| \| **Gene** \| \| --- \| | **Protein*** | | **5'-Forward Primer-3'** | **5'-Reverse Primer-3'** |
| --- | --- | --- | --- | --- | --- |
| *Actb* | | β-actin | CGATGCCCTGAGGCTCTTT | TGGATGCCACAGGATTCCA |
| *Bgn* | | BGN | TCAACAACCCTGTGCCCTAC | GGCAACCACTGCCTCTACTT |
| *Ccl4* | | CCL-4 | TCTGTGCAAACCTAACCCCG | GAGGGTCAGAGCCCATTGGT |
| *Ccr2* | | CCR-2 | GCCATCATAAAGGAGCCATACCT | TGTGGTGAATCCAATGCCCT |
| *Cd68* | | CD68 | TGTCTGATCTTGCTAGGACCG | GAGAGTAACGGCCTTTTTGTGA |
| *Cd74* | | CD74 | CCTTGCTGATGCGTCCAATG | CTGGGTCATGTTGCCGTACT |
| *Col1a1* | | COL1A1 | AGGTATGCTTGATCTGTAT | CAGTCCAGTTCTTCATTG |
| *Col1a2* | | COL1A2 | TTCACCTACTCTGTCCTA | CGAGATGGCTTATTTGTTT |
| *Col3a1* | | COL3A1 | GAAAGTGAGGGAAGCCAAA | TCAGAGTAGCACCATCAGT |
| *Cygb* | | CYGB | TGCTCATGGAGAAAGTGCCG | GTCCTCGCAGTTGGCATACA |
| *Cyp7a1* | | CYP7A1 | AGCAACTAAACAACCTGCCAGTACTA | GTCCGGATATTCAAGGATGCA |
| *Cyp8b1* | | CYP8B1 | AGTACACATGGACCCCGACATC | GGGTGCCATCCGGGTTGAG |
| *Dgat2* | | DGAT2 | GGAATAAGTGGGAACCAGATCA | CCGCAAAGGCTTTGTGAAG |
| *Gapdh* | | GAPDH | TGTGTCCGTCGTGGATCTGA | CCTGCTTCACCACCTTCTTGAT |
| *H2aa* | | H2Aa | GAGGAGCCGGTTCTGAAACA | TCGCAGGCCTTGAATGATGA |
| *H2ab1* | | H2-Ab1 | GTGTACCAGTTCATGGGCGA | ACGTACTCCTCCCGGTTGTA |
| *H2dma* | | H2-DMa | AGAGAATGCCCTGTGTGGTG | GGTAGTCACGAGATGCCTGG |
| *H2dmb1* | | H2-DMb1 | ACTGCCTCTATTCAGGTCCCC | CTTGAACCCCCACCTCATTTCA |
| *H2eb1* | | H2-Eb1 | CTGTCACGGTCGAGTGGAAA | CCTGTTGGCTGAAGTCCAGA |
| *Hmgcr* | | HMG-CoA R | GATGATTATGTCTTTAGGCTTG | CAGAGAGAAACACTTGGT |
| *Idol* | | IDOL | AGGAGATCAACTCCACCTTCTG | ATCTGCAGACCGGACAGG |
| *Ifr8* | | IFR8 | GACAGGGTCGACAGAGTGTG | CTCCTGCTTTGACTGAGGGG |
| *Loxl2* | | LOXL2 | AACTATGAAGTGCCAGAATC | TACATCCAGATGCGGTAG |
| *Lrp1* | | LRP-1 | AAGACAATGCTACAGACT | TGTTGAAGACCTTGATGT |
| *Lum* | | LUM | TGAATGTATGTGCGTTCT | GGATGTCATAATCGTAGTATTG |
| *Ccl2* | | MCP-1 | TTCCTCCACCACCATGCAG | CCAGCCGGCAACTGTGA |
| *Mttp* | | MTP | CTACCAGGCCCAACAAGAC | CGCTCAATTTTGCATGTATCC |
| *Pcsk9* | | PCSK9 | AGGTGGAGGTGTATCTCTTAGA | CACTTGCTCGCCTGTCTG |
| *Rplp0* | | RPLP0 (36B4) | CCTGAAGTGCTCGACATCAC | CCACAGACAATGCCAGGAC |
| *Saa1* | | SAA | CCAATTACTACAGACCTCC | AGCATCTTCAGTGTTCCTA |
| *Saa2* | | SAA-2 | GTTCAGAAGGCTGTGTTGGGG | CCCAGAGAGCATCTTCAGTGTT |
| *Soat2* | | SOAT-2 | CCCTATGGGAGGGCTATGC | TAACTCTTGTGGTCTTGCTTGTA |
| *Srebp2* | | SREBP-2 | GTGTGCGGAGGAGAAAATCC | GTGTGCGGAGGAGAAAATCC |
| *Tgfb1* | | TGFB1 | CGCAACAACGCCATCTAT | TGCTTCCCGAATGTCTGA |
| *Tgfbi* | | TGFBI | GTTCACCATGGACCGGATGT | GGCCACCAGCATGCTAAAAC |
| *Tgfb1* | | TGFβ | CGCAACAACGCCATCTAT | TGCTTCCCGAATGTCTGA |
| *Tlr2* | | TLR-2 | CAGTCTTCCTAGGCTGGTGC | AAGGAAACAGTCCGCACCTC |
| *Tlr4* | | TLR-4 | CCTGCATAGAGGTAGTTC | TGGTTGAAGAAGGAATGT |
| *Tnf* | | TNF-α | GGCTGCCCCGACTACGT | ACTTTCTCCTGGTATGAGATAGCAAAT |

*Abbreviations used: β-actin, beta actin; BGN, biglycan; CCL-4, C-C motif chemokine 4; CCR-2, C-C chemokine receptor type 2; CD68, cluster of differentiation 68; CD74, cluster of differentiation 74; COL1A1, collagen alpha-1 (I) chain; COL1A2, collagen alpha-2(I) chain; COL3A1, collagen alpha-1(III) chain; CYGB, cytoglobin; CYP7A1, cholesterol 7 alpha-hydroxylase 1; CYP8B1, cytrochrome P450, family 8, subfamily B, polypeptide 1; DGAT, diacylglyceride transferase; GAPDH, glyceraldehyde 3-phosphate dehydrogenase; H2-Aa, H-2 class II histocompatibility antigen, A-B alpha chain; H2-Ab1, H-2 class II histocompatibility antigen, A beta chain; H2-DMa, H2 Class II histocompatibility antigen, M alpha chain; H2-DMb1, H2 Class II histocompatibility antigen, M beta 1 chain; H2-Eb1, H-2 class II histocompatibility antigen, E-B beta chain; HMGCR, 3-hydroxy-3-methyl-glutaryl-coenzyme A reductase; IDOL, inducible degrader of low-density lipoprotein receptor; IFR-8, interferon regulatory factor 8; LOXL-2, lysyl oxidase homolog 2; LRP-1, low density lipoprotein receptor related protein 1; LUM, lumican; MCP-1, monocyte chemoattractant protein 1; MTP; microsomal transfer protein; PCSK9, proprotein convertase subtilisin/kexin type 9; RPLP0 (36B4), ribosomal protein, large, P0; SAA, serum amyloid A; SAA-2, serum amyloid A2; SOAT-2, sterol O-acyltransferase 2; SREBP-2, sterol regulatory element-binding protein 2; TGFB1, transforming growth factor beta 1; TGFBI, transforming growth factor beta induced; TLR-2, toll like receptor-2; TLR4, toll-like receptor 4; TGFβ, transforming growth factor β, TNF-α, tumor necrosis factor alpha.

**Table S5. Fecal neutral sterols of *Ldlr*^-/-^ mice after 14 weeks of diets**

| **Variable** | **Chow** | **HFD + Vehicle** | **HFD + L654** |
| --- | --- | --- | --- |
| Cholesterol (ng/g) | 23684 ± 4273 | 85085 ± 6863* | 129721 ± 13169*# |
| Coprostanol (ng/g) | 15343 ± 2887 | 125050 ± 13193* | 63361 ± 12440*# |
| Coprostanone (ng/g) | 606 ± 384 | 8012 ± 1773* | 5221 ± 1460* |
| Total neutral sterols (ng/g) | 39634 ± 6707 | 218147 ± 16422* | 198303 ± 12793* |
| Cholesterol/coprostanol ratio | 1.62 ± 0.24 | 0.75 ± 0.10* | 3.20 ± 0.97# |

Values are mean ± SEM for all groups (Chow, *n* = 5/group; HFD + Vehicle and L654, *n* = 9/group). *****indicates *p*-value < 0.05 vs. Chow reference group**. #**indicates *p*-value < 0.05 vs. HFD +Vehicle control.

**Table S6. Body and tissue weights of *Apoe*^-/-^ mice on chow diet after 7 weeks of I.P. injections**

| **Variable** | **Vehicle** | **L567** | **L654** |
| --- | --- | --- | --- |
| Body Weight (g) | 30.6 ± 0.61 | 30.4 ± 0.66 | 30.4 ± 0.49 |
| Liver Weight (g) | 1.39 ± 0.06 | 1.39 ± 0.06 | 1.35 ± 0.04 |
| % Liver | 4.48 ± 0.12 | 4.67 ± 0.09 | 4.39 ± 0.09 |
| Epididymal Adipose (g) | 0.54 ± 0.06 | 0.47 ± 0.06 | 0.62 ± 0.06 |
| % Epididymal Adipose | 2.09 ± 0.25 | 1.70 ± 0.24 | 1.99 ± 0.15 |

Values are mean ± SEM for all groups (*n* = 15/group).

**Table S7. Fecal bacterial glycine lipids of *Apoe*^-/-^ mice on chow diet after 7 weeks of I.P. injections**

| **Variable** | **Vehicle** | **L567** | **L654** |
| --- | --- | --- | --- |
| Total glycine lipids (ng/g) | 16147 ± 2358 | 15368 ± 2685 | 16218 ± 1185 |
| L1256 (ng/g) | 8805 ± 844 | 8722 ± 1334 | 11256 ± 599 |
| L654 (ng/g) | 258 ± 250 | 288 ± 280 | 1638 ± 841 |
| L567 (ng/g) | 1423 ± 218 | 1447 ± 465 | 1441 ± 297 |
| L430 (ng/g) | 4373 ± 2643 | 3840 ± 1258 | 710 ± 332 |
| L342 (ng/g) | 1288 ± 292 | 1073± 539 | 1174 ± 47 |

Values are mean ± SEM for all groups (*n* = 4/group).
